# Metformin With or Without Clomiphene Citrate Versus Laparoscopic Ovarian Drilling With or Without Clomiphene Citrate to Treat Patients With Clomiphene Citrate-Resistant Polycystic Ovary Syndrome: A Systematic Review and Meta-Analysis

**DOI:** 10.3389/fphar.2022.576458

**Published:** 2022-06-22

**Authors:** Ming-Li Sun, Wen-Pei Bai, Qing-Kun Song, Hui-Ying Wang, Guo-Lan Gao, Liang Zheng, Xing-He Wang

**Affiliations:** ^1^ Phase I Clinical Trial Center, Beijing Shijitan Hospital Affiliated to Capital Medical University, Beijing, China; ^2^ Department of Gynecology, Beijing Shijitan Hospital Affiliated to Capital Medical University, Beijing, China; ^3^ Department of Science and Technology, Beijing Shijitan Hospital Affiliated to Capital Medical University, Beijing, China; ^4^ Department of Gynecology, Shenzhen Hospital Affiliated to Savaid Medical School, University of Chinese Academy of Sciences, Shenzhen, China; ^5^ Research Center for Translational Medicine, Shanghai East Hospital, Tongji University School of Medicine, Shanghai, China

**Keywords:** metformin, laparoscopic ovarian drilling, clomiphene citrate resistance, live-birth, meta-analysis

## Abstract

**Introduction:** Which is optimal to treat clomiphene citrate-resistant polycystic ovary syndrome (CCR-PCOS) with LOD or metformin remains a problem. There are three inconsistent or even contradictory views.

**Objectives:** The present meta-analysis aimed to evaluate the effectiveness and safety of Metformin with or without CC and to compare them with LOD with or without CC (Met/Met-CC vs. LOD/LOD-CC) in women with CCR-PCOS who also have anovulation.

**Data source:** The PubMed, Cochrane, and Embase databases were searched to identify relevant studies reported between 1 Jan 1966 and 31 Aug 2019; the search was updated on 17 May 2022.

**Study eligibility criteria:** We included randomized controlled trials (RCTs) of CCR-PCOS that had considered Met/Met-CC and LOD/LOD-CC as the exposure variables and fertility as the main outcome variable.

**Study appraisal and synthesis methods:** We assessed study quality using the Cochrane risk-of-bias tool. The primary effectiveness outcome was live birth/ongoing pregnancy rate and the primary safety outcome was miscarriage rate. A fixed-effect meta-analysis was performed. The robustness of the results was assessed using sensitivity analyses. Meta-regression and subgroup analysis were performed to examine the reasons for heterogeneity. Publication bias was examined using the funnel plot, Egger linear regression, and Begg rank correlation tests. The quality of this meta-analysis was estimated according to the GRADE approach. This meta-analysis has been registered in PROSPERO (CRD42021240156).

**Results:** Among 71 potentially relevant studies, we included five RCTs in our meta-analysis. We found no difference in effectiveness between Met-CC and LOD in terms of live birth/ongoing pregnancy (RR = 1.02, 95% CI: 0.87–1.21, z = 0.28; *p* = 0.780), and miscarriage rates (RR = 0.79, 95% CI: 0.46–1.36, z = 0.86; *p* = 0.390). I2 tests results revealed moderate or no heterogeneity (I2 = 51.4%, *p* = 0.083; I2= 0.0%; *p* = 0.952). Sensitivity analysis confirmed the robustness of the results. Funnel plot, Egger linear regression, and Begg rank correlation tests implied no publication bias (*p* > 0.05). LOD was more expensive than Met (€1050 vs. €50.16). The evidence quality was moderate.

**Conclusion:** There is no evidence on the difference in the outcomes between the two interventions regarding ovulation, pregnancy, and live birth. As LOD is an invasive procedure and carries inherent risks, the use of Met/Met-CC should be the second-line treatment for women with CCR-PCOS.

**Systematic Review Registration:** identifier CRD42021240156.

## Introduction

Polycystic ovary syndrome (PCOS), also known as Stein–Leventhal syndrome, was originally described by Stein and Leventhal in 1935. It is characterized by amenorrhea or occasional menometrorrhagia, hirsutism, infertility, and large, pale, polycystic ovaries with thickened capsules (Stein and Leventhal, 1935; Azziz and Adashi, 2016; Greenwood et al., 2018). According to the Rotterdam consensus criteria, PCOS can only be diagnosed when other androgen excess disorders have been excluded and at least two of the following three criteria are present: oligo-ovulation or anovulation, biochemical and/or clinical signs of hyperandrogenism, and polycystic ovaries identified under ultrasound examination (Escobar-Morreale, 2018; Chan et al., 2017; Engmann et al., 2017). PCOS is the most common endocrine disorder, affecting 6%–21% of women of reproductive age (He et al., 2019; Izadi et al., 2019; Makrinou et al., 2020). Approximately 75% of women with PCOS suffer infertility due to anovulation (Costello et al., 2019). One of the primary pharmacological agents used to treat this condition is clomiphene citrate (CC) (Schroeder and American College of Obstetricians and Gynecologists, 2003; Legro et al., 2013; Legro et al., 2014; Amer et al., 2017; Kar, 2012; Bayar et al., 2006; NHRMC, 2018; Teede et al., 2018; Wang et al., 2019; Franik et al., 2018) which induces ovulation in 75%–80% of patients with infertility due to PCOS (Messinis, 2005; Birch Petersen et al., 2016).

Nevertheless, some patients with PCOS show CC resistance (CCR), defined as failure to achieve ovulation after the dose of CC has been gradually increased to 150 or 250 mg/day—the final dosage differed among the studies—in at least three consecutive cycles (Malkawi et al., 2003; Palomba et al., 2004; Palomba et al., 2005; Ashrafinia et al., 2009; Hamed et al., 2010; Palomba et al., 2010; Abu Hashim et al., 2011; Elgafor el sharkwy, 2013). In a previous review, Bordewijk et al. concluded that LOD with or without medication-induced ovulation may result in lower live birth rates than medication-induced ovulation alone in women with anovulatory CCR-PCOS (Bordewijk et al., 2020a). Another review by Yu et al. concluded that no recommendation could be made regarding ovulation induction in patients with CCR-PCOS because the available studies presented low-quality evidence and wide confidence intervals (Yu et al., 2017). The new international evidence-based guideline for PCOS recommended both laparoscopic ovarian drilling (LOD) and combined metformin and CC (Met-CC) as second line therapies to treat CCR-PCOS, without defining any treatment timeline (NHRMC, 2018; Teede et al., 2018).

Prominently, LOD is an invasive procedure and carries inherent risks. Conversely, drug safety is always the center of attention. Metformin has been widely used clinically for >60 years and there is sufficient evidence of its safety and tolerability in most populations (Lv and Guo, 2020). Pregnant and lactating women are considered “therapeutic orphans” because they generally have been excluded from the clinical drug study and new drug development process owing to safety, legal, and ethical concerns. Most medications prescribed for pregnant and lactating women are “off-label” because most of the clinically approved medications do not have appropriate drug labeling information (Ren et al., 2021). The limited evidence indicates the long-term safety of the fetus exposed to metformin excluding mild adverse anthropometric profiles (sex hormone binding globulin levels and long-term body mass index in offspring) (Roy and Sahoo, 2021; Zhu et al., 2022). Although the FDA has not completely ruled out the risk of metformin in pregnancy, “these studies cannot establish the lacking metformin-associated risk because of methodological limitations, including the small sample size and inconsistent comparator groups” (https://packageinserts.bms.com/pi/pi_glucophage.pdf), many of the reviewed studies concluded that metformin is a safe choice at the beginning of pregnancy without persuasive evidence of increased risk for miscarriages or congenital malformations (Quadir, 2021).

In the present study, we conducted a systematic review and meta-analysis of published randomized controlled trials (RCTs) to evaluate the effectiveness and safety of Met, Met-CC, LOD, and LOD combined with CC (LOD-CC), as well as to compare Met/Met-CC (Met with or without CC) with LOD/LOD-CC (LOD with or without CC) in patients with CCR-PCOS who showed anovulation-related infertility. In so doing, we sought to establish which approach should be used as the primary treatment and to confirm which of the above three viewpoints is correct.

## Materials and Methods

The present meta-analysis was performed according to the Preferred Reporting Items for Systematic Reviews and Meta-Analysis (PRISMA) statement protocol (Higgins et al., 2011a) (Supplementary Appendix S1), and was registered in the PROSPERO (Registration number: CRD42021240156).

### Information Sources and Search Strategy

PubMed, Embase, and the Cochrane Central Register of RCTs were searched for articles published between 1 Jan 1966 and 31 Aug 2019. In accordance with the PICOS strategy, search terms were formulated as follows: 1) patient population: “polycystic ovarian syndrome”, “polycystic ovary syndrome”, “polycystic ovarian disease”, “PCOS”, and “PCOD”; 2) intervention: “metformin”, “dimethylguanylguanidine”, “dimethylbiguanidine,” “glucophage”, “dimethylbiguanide”, and “DMBG”; 3) control: “laparoscopic ovarian drilling”, “laparoscopic ovarian diathermy”, “LOD”, “laparoscopic ovarian electrocautery”, and “LOE”; 4) outcomes: “ovulation”, “pregnancy”, and “live birth”; 5) study design: “randomized controlled trial”, “random allocation”. Results were restricted to humans and no language restrictions were applied (Supplementary Appendix S2). The reference lists of the included articles were scanned for additional relevant studies. Grey (unpublished) literature was identified by searching the websites of clinical practice guideline collections, clinical trial registries, national and international medical specialty societies, and recent conference abstracts. Full-text articles of potentially relevant studies that were unavailable through the university library were requested from the authors. The literature search was updated on 17 May 2022. The search strategies were formulated by physicians (M-LS and X-HW), gynecologists (G-LG and W-PB), and statisticians (LZ and Q-KS).

### Eligibility Criteria and Study Selection

Two reviewers (ML Sun and L Zheng) independently screened the titles and abstracts to determine whether the articles were relevant to the meta-analysis based on the pre-defined inclusion criteria listed below. The full texts of potentially eligible studies were then reviewed before the final selection. Any disagreements were resolved in consultation with the principal investigator (XH Wang).

The inclusion criteria were as follows: 1) study population of patients with both CCR-PCOS and anovulation-related infertility; 2) intervention of LOD-controlled Met treatment despite continuing CC; 3) reporting of fertility outcomes (ovulation, pregnancy, and live-birth rates); 4) RCT study design. The exclusion criteria were as follows: 1) duplicates; 2) study design other than RCT (e.g., reviews, meta-analyses, case reports, guidelines, trial protocols); 3) absence of comparison between Met/Met-CC and LOD/LOD-CC; 4) absence of fertility outcomes.

### Data Extraction

Data were extracted from the included articles by two independent reviewers (M-LS and LZ) using standardized data extraction sheets. Any disagreements were resolved in consultation with the principal investigator (X-HW). If available, the following information was extracted from each article: study period, inclusion, and exclusion criteria, first author, year of publication, subjects’ country of residence, definition of CCR, number of participants in each group, clinical characteristics of the participants, treatment regimens, duration of treatment, follow-up period, and fertility outcomes (ovulation, pregnancy, and live-birth rates). Adverse events (AEs), including miscarriage, multiple pregnancy, ectopic pregnancy, ovarian hyperstimulation syndrome (OHSS), drug-related AEs, and intra- or post-operative complications, were identified according to the data originally documented in the articles.

### Assessment of Risk of Bias

Two researchers (M-LS and LZ) independently conducted quality assessment of all the included articles using the Cochrane risk-of-bias tool in the following domains: selection bias, performance bias, detection bias, attrition bias, reporting bias, and other bias (Higgins et al., 2011a; Higgins et al., 2011b). Each domain was classified as low, unclear, or high risk. If there were discrepancies, the final assessment decision was made in consultation with the principal investigator (X-HW). The overall quality of this meta-analysis was estimated according to the GRADE four-step approach (Higgins et al., 2011b).

### Data Synthesis

The overall meta-analysis was performed following the appropriate Cochrane Guidelines (Higgins et al., 2011a). The primary effectiveness outcome was live birth/ongoing pregnancy and the primary safety outcome was miscarriage. The secondary effectiveness outcomes were pregnancy and ovulation-induction, while the secondary safety outcomes included multiple pregnancy, ectopic pregnancy, OHSS, medical-related AEs and surgical complications. We also compared the costs of the two treatments. Effect size was calculated based on the risks (relative risk; RR) provided by each study. A fixed-effect meta-analysis was then conducted (Higgins et al., 2011a).

### Investigation of Heterogeneity

Heterogeneity among the included studies was analyzed using the *I* ^2^ test (Higgins et al., 2011a) as follows: *I* ^2^ = [(Q–df)/Q] × 100%, where Q is the *χ* ^2^ heterogeneity statistic and df is the degrees of freedom. *I* ^2^ values >75% indicate high heterogeneity, whereas values between 50% and 75% indicate moderate heterogeneity. *I* ^2^ values between 25% and 50% indicate low heterogeneity, and values below 25% indicate no heterogeneity.

### Sensitivity Analysis

We conducted sensitivity analyses of the primary effectiveness outcome (live birth/ongoing pregnancy), to determine whether the conclusions were robust to arbitrary decisions made about eligibility, and analysis. Sensitivity analysis was performed using the “random-effects model” and “leave-one-out” methods.

### Meta-Regression Analysis

If any heterogeneity occurred, the reasons for it were ascertained using a meta-regression analysis of the primary effectiveness outcome (live birth/ongoing pregnancy).

### Subgroup Analysis

Subgroup analysis of the primary effectiveness outcome (live birth/ongoing pregnancy) was also performed to explain expected significant heterogeneity.

### Assessment of Reporting Biases

To reduce reporting bias, we were alert to data duplication and ensured that our search for eligible studies was comprehensive. Potential publication bias was examined using the funnel plot, Egger linear regression, and Begg rank correlation tests (Higgins et al., 2011a).

### Overall Quality of the Body of Evidence: “Summary of Findings” Table

We generated a “Summary of findings” table using GRADEPro 3.6 software. This table shows the overall quality of the evidence for the main review outcomes according to the GRADE criteria. We justified judgements about evidence quality (high, moderate, low, or very low), and documented and incorporated these judgements into the reporting of results for each outcome.

### Statistics and Statistical Software

The statistical significance level was set at *p* < 0.05. In the meta-analysis, Review Manager 5.3 and one of the Cochrane Collaboration Tools were used to create the risk-of-bias graph; the statistical software package Stata16 (Stata Corp., College Station, TX, United States), and GRADEpro 3.6 software were also used.

## Results

### Study Selection

**FIGURE 1:**
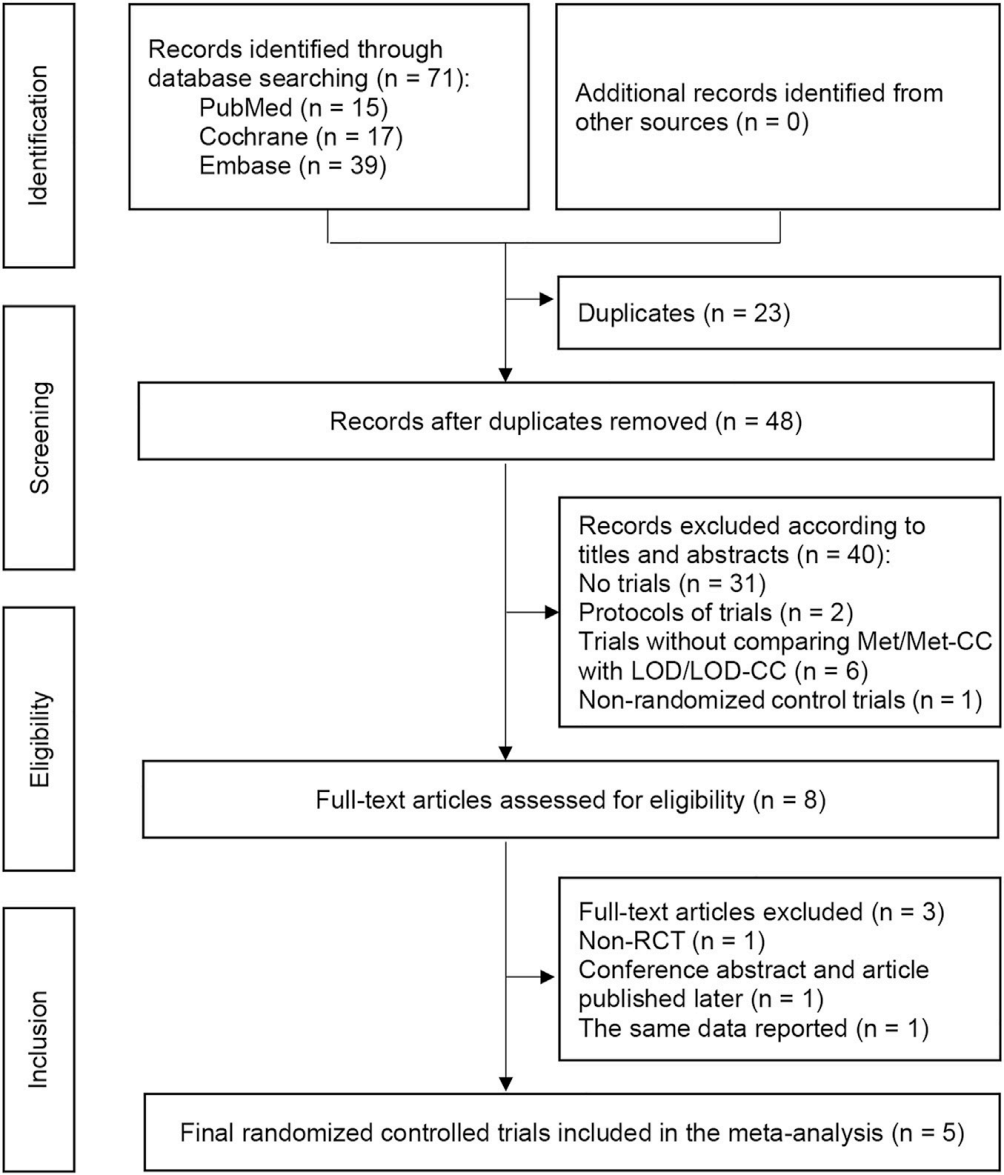
Preferred Reporting Items for Systematic Reviews and Meta-analyses flow chart depicting the process of paper selection and the number of papers in each phase. Notes: RCT, randomized controlled trial.

Overall, 71 studies were retrieved from the electronic databases. Twenty-three duplicates were removed, resulting in 48 unique titles. Following title and abstract review, we assessed eight full-text articles for eligibility. Five RCTs (Palomba et al., 2004; Palomba et al., 2005; Hamed et al., 2010; Palomba et al., 2010; Abu Hashim et al., 2011) met all the inclusion and exclusion criteria, and were thus included in our main meta-analysis (Figure 1). In the 2010 study by Hamed et al., ovulation induction, pregnancy, and first trimester abortion rate were reported, rather than live-birth rate, so we used the ongoing pregnancy rate in the analysis of live-birth rate (Abu Hashim et al., 2011).

### Study Characteristics

The characteristics of the included RCTs are summarized in Table 1. The studies involved women with CCR-PCOS from two countries: Egypt and Italy.

**TABLE 1:** Characteristics of the included randomized controlled trials.

Study, authors (yr)	Study country	Treatment	Treatment regimens^#^	Duration of treatment^&^	The follow-up period	Frequency of intercourse (advice from researcher)	Time of pregnancy after treatment
Hashim HA. (2011)	Egypt	1) Met-CC	1) Met 500 mg tid for 6–8 weeks; then CC 100 mg for 5 days starting from day 3 of menstruation; increased by 50 mg for the next cycle. 2) LOD operation. 3) HCG was given when one follicle measuring at least 18 mm was found	1) 6 cycles	1) six cycles. 2) 6 months. 3) until the end of the pregnancy	Patients with ovulation to have intercourse naturally or 24–36 h after hCG injection	NR
		2) LOD		2) one LOD			NR
Hamed et al. (2010)	Egypt	1) Met	1) Met 850 mg bid after dL. 2) LOD operation	1) 6 cycles or 30 weeks	1) six cycles or 30 weeks 2) 6 months. 3) until 13 weeks of gestation	To have Intercourse every other day when a mature follicle was seen on ultrasound	NR
		2) LOD		2) one LOD			NR
Palomba et al. (2010)	Italy	1) Met-CC	1) Met 850 mg qd started from day 1 of a progesterone-induced withdrawal bleeding, and the dosage was increased after 1 week up to 850 mg bid. 2) LOD operation	1) 6 cycles	1) six cycles. 2) six cycles. (3) until the end of the pregnancy	To have Intercourse once per 3 days starting on day 9 after uterine bleedings	NR
		(2) LOD		2) one LOD			NR
Palomba et al. (2005)	Italy	1) Met-CC	1) Met 850 mg bid after dL; placebo 1 tablet bid after LOD. 2) CC 150 mg qd for 5 days (the 3rd - 7th day of progesterone-induced uterine bleeding)	1) 6 months	1) 6 months. 2) 6 months. (3) until the end of the pregnancy	To have inter-course 4 times/2 days from the time of a follicle with Ø ≥18 mm	7 (range 1–15) months$
		2) LOD-CC		2) one LOD			6 (range 3–13) months$
Palomba et al. (2004)	Italy	1) dl-Met	Met 850 mg bid after dL; Multivitamins 1 tablet bid after LOD.	1) 6 months 2) one LOD	1) After the study, a 6-months extension of the follow-up period was done for each treatment group. 2) until the end of the pregnancy	To have inter-course/2 days for 4 times from the time of a follicle with Ø ≥18 mm	NR
		2) LOD-placebo					NR

Study, authors (yr)	Groups	Definition of CCR (max dose of CC, mg/day)	Diagnosis of ovulation (progesterone level, ng/ml)	Full analysis set sample size (n)	Per protocol set sample size (n)	Clinical characteristics of participants		Fertility outcome (Rate, %)		
						Age of patients in each group (yrs)	BMI (kg/m^2^)	Ovulation	Pregnancy	Live-birth or ongoing pregnancy^*^
Hashim HA, et al. (2011)	1) Met-CC	150	5	138	138	27.2 ± 2.5	26.2 ± 3.4	68.8 (95/138)	64.5 (89/138)	58.7 (81/138)
	2) LOD			144	144	26.5 ± 2.3	26.1 ± 3.5	71.5 (103/144)	66.0 (95/144)	59.7 (86/144)
Hamed et al. (2010)	1) Met	150	5	55	55	23.6 ± 2.6	35.6 ± 4.4	58.2 (32/55)	20.0^@^ (11/55)	16.4* (9/55)
	2) LOD			55	55	24.3 ± 4.5	36.1 ± 3.6	76.4 (42/55)	38.2 (21/55)	30.9^*^ (17/55)
Palomba et al. (2010)	1) Met-CC	250	10	25	23	27.5 ± 4.8	30.2 ± 3.4	80 (20/25)	56.0 (14/25)	48.0 (12/25)
	2) LOD			25	24	28.2 ± 4.3	29.8 ± 3.2	68 (17/25)	60.0 (15/25)	52.0 (13/25)
Palomba et al. (2005)	1) Met-CC	150	10	8	8	27.2 ± 2.2	28.4 ± 1.6	87.5 (7/8)	75.0 (6/8)	50.0 (4/8)
	2) LOD-CC			20	20	25.4 ± 2.4	27.7 ± 1.8	70 (14/20)	60.0 (12/20)	35.0 (7/20)
Palomba et al. (2004)	1) dl-Met	150	10	60	54	26.8 ± 2.2	28.1 ± 1.7	70 (42/60)	18.57 (39/60)	53.3 (32/60)
	2) LOD-placebo			60	55	27.5 ± 2.4	27.6 ± 1.6	58.3 (35/60)	13.42 (31/60)	33.3 (20/60)

**Study, authors (yr)**	**Groups**	**Multiple pregnancy (%)**	**Ectopic pregnancy (%)**	**Miscarriage (%)**	**OHSS**	**Drug-related AEs and intra- or post-operative complications**	**Costs**
Hashim HA. (2011)	1) Met-CC	2.9 (4/138)	NR	5.8 (8/138)	0/138	(1) 9.4% (13/138) of patients in Met-CC group suffered gastrointestinal side effects (mainly nausea and vomiting), but continued therapy. (2) LODs were completed successfully without intra- or post-operative complications	NR
	2) LOD	0/144	NR	6.3 (9/144)	0/144		NR
Hamed et al. (2010)	1) Met	NR	NR	3.6^@^ (2/55)	NR/55	(1) 18.2% (10/55) of patients in Met group reported self-limiting dizziness, nausea, and flatulence, but continued therapy. (2) LODs were completed successfully without surgical complications	NR
	2) LOD	NR	NR	7.3^@^ (4/55)	NR/55		NR
Palomba et al. (2010)	1) Met-CC	0/25	NR	8 (2/25)	NR/25	(1) 17.4% (4/23) of patients in Met-CC group reported diarrhea, abdominal pain, hot flashes, and nausea, but continued therapy. (2) LOD was not performed in 1 obese woman. (3) 8% (2/25) cases: a minimal endometriosis and avascular pelvic adhesions	NR
	2) LOD	0/25	NR	8 (2/25)	NR/25		NR
Palomba et al. (2005)	1) Met-CC	0/8	NR	25 (2/8)	NR/8	(1) Minor gastrointestinal symptoms, with no difference in AEs between 2 groups (12.5% vs 15.0%, *p* = 1·00). (2) No CC-related AEs were observed. (3) No intra- or post-operative complications	NR
	2) LOD-CC	0/20	NR	25 (5/20)	NR/20		NR
Palomba et al. (2004)	1) dl-Met	0/60	NR	10 (6/60)	NR/60	(1) 22.2% (12/54) patients in dL-Met group reported diarrhea, flatulence, and nausea; (2) 5.5% (3/55) in LOD-placebo group reported gastralgia and constipation. (3) No intra- or post-operative complications	50.16€ for Met
	2) LOD-placebo	0/60	NR	15 (9/60)	NR/60		1050 € for LOD

Notes: Met, metformin; CC, clomiphene citrate; LOD, laparoscopic ovarian drilling/diathermy; dL, diagnostic laparoscopy; AEs, adverse events; SAE, serious adverse events. tid, thrice daily; bid, twice daily; qd, once daily. HCG, human chorionic gonadotropin injection. P, progesterone. yr, year. NR, not reported; OHSS, ovarian hyperstimulation syndrome. ^#^, all treatments were suspended in patients who conceived. ^&^, Those who conceived were observed until the end of the pregnancy (for up to further 9 months) to obtain live birth data for each treatment arm, except the study of Hamed HO, et al. (2010). ^*^, ongoing pregnancy rate was calculated based on first trimester abortion. ^$^, median time. ^@^, first trimester miscarriage rate.

The included trials had a total of 590 subjects (from 28 to 282 in each study), of which 115 were treated using Met, 171 using Met-CC, 284 using LOD, and 20 using LOD-CC (Palomba et al., 2004; Palomba et al., 2005; Hamed et al., 2010; Palomba et al., 2010; Abu Hashim et al., 2011). Patients were treated using Met for six cycles or followed-up for 6 months or 30 weeks after LOD. Ovulation was identified when the serum progesterone level was ≥5 ng/mL (Hamed et al., 2010; Abu Hashim et al., 2011) or ≥10 ng/ml (Palomba et al., 2004; Palomba et al., 2005; Palomba et al., 2010). Pregnancy was identified based on an increase in β-human chorionic gonadotropin or sonographic evidence of an intrauterine gestational sac. In most of the included studies, subjects who conceived were observed until the end of pregnancy (for up to a further 9 months) to obtain live-birth data for each treatment arm (Palomba et al., 2004; Palomba et al., 2005; Palomba et al., 2010; Abu Hashim et al., 2011) although the study by Hamed et al. only followed up the pregnancy until 13 weeks’ gestation to obtain first trimester abortion and ongoing pregnancy data (Abu Hashim et al., 2011). Ovulation induction, pregnancy, live birth/ongoing pregnancy, and miscarriage rates were calculated as the number of women with ovulation, pregnancy or living baby, and abortion, respectively, divided by the total number of subjects randomized in the same group (See Table 1).

### Risk of Bias

**FIGURE 2:**
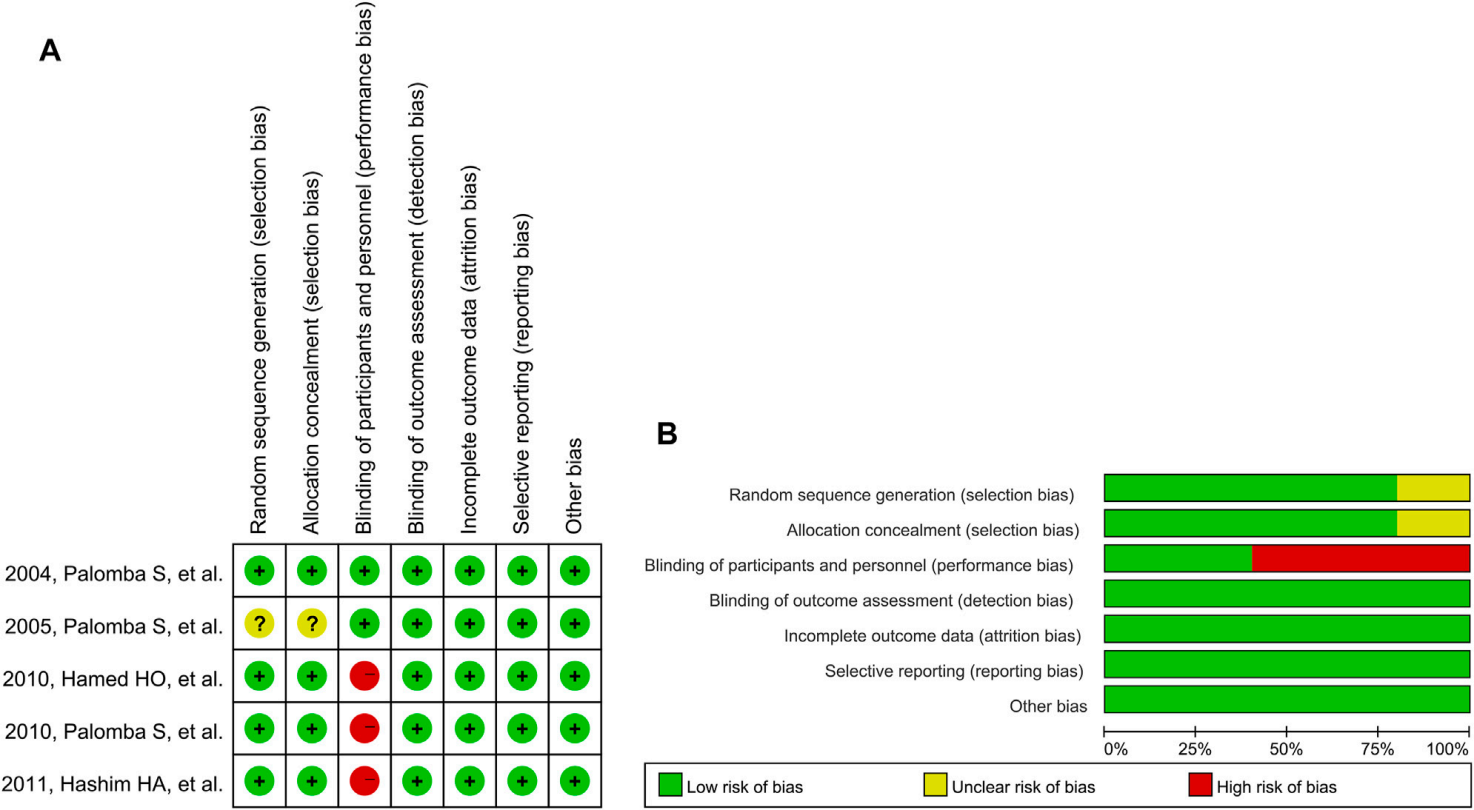
Risk of bias graph for reviewing authors’ judgements: **(A)** Individual studies, and **(B)** All studies. Note: The colors have the same meaning in both **(A)** and **(B)**.

The results of the quality assessment are shown in Figure 2. In “randomization and allocation concealment” category, four studies (Palomba et al., 2004; Hamed et al., 2010; Palomba et al., 2010; Abu Hashim et al., 2011) had low risk of bias and one (Palomba et al., 2005) had unclear risk of bias because the randomization and allocation concealment protocols were described unclearly. Although none of the studies described blinding of outcome assessment clearly, a low risk of detection bias was granted to all of them because the outcomes ware objective indicators. The first three studies had a high risk of performance bias because their trial protocols did not involve dual-mode analogue (Hamed et al., 2010; Palomba et al., 2010; Abu Hashim et al., 2011).

### Synthesis of Results

To compare Met/Met-CC with LOD/LOD-CC in terms of both effectiveness on fertility outcomes and safety, we pooled data from all subjects in the included trials using the fixed-effects model.

**FIGURE 3:**
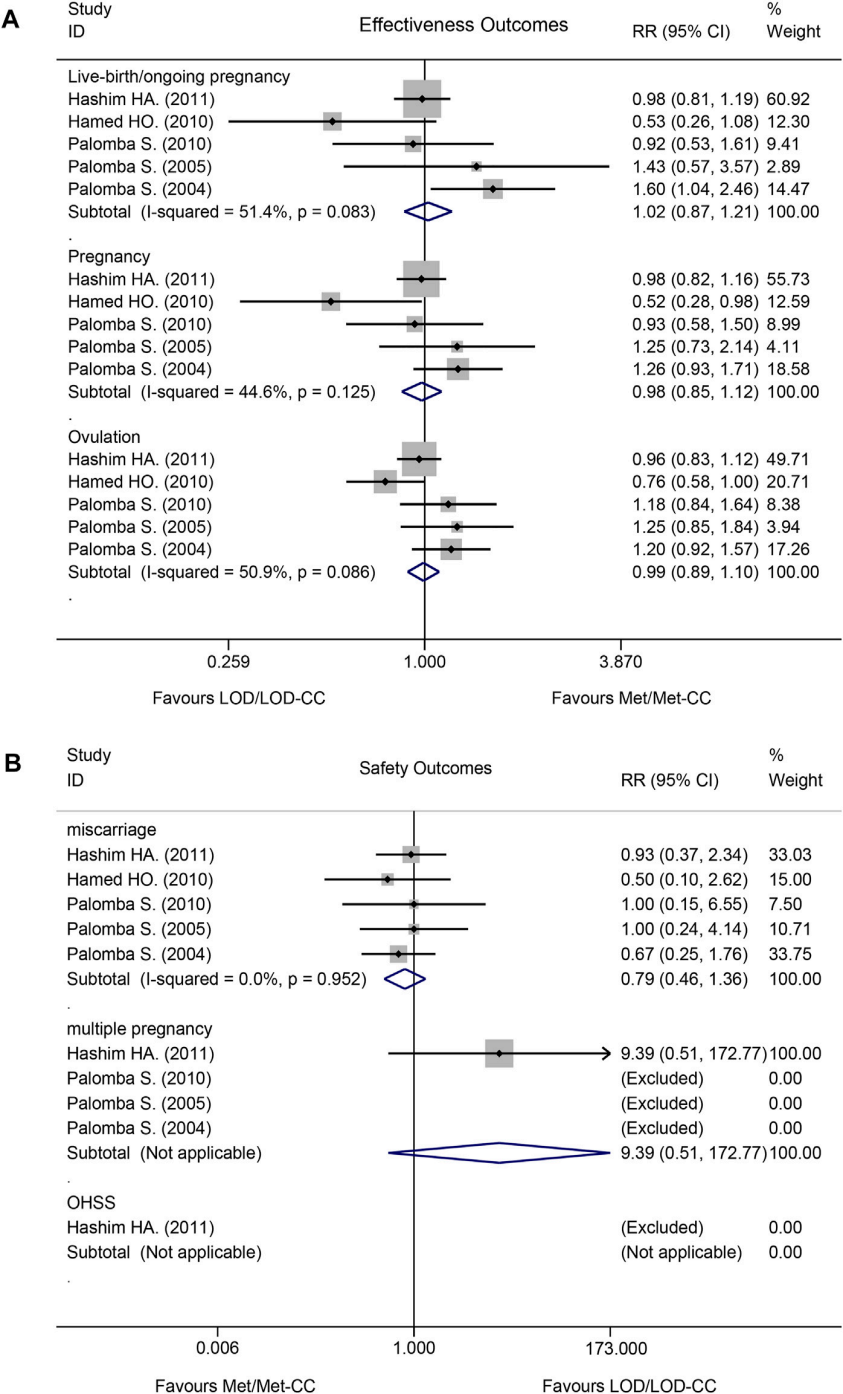
Forest plot of effectiveness and safety outcomes in the Met/Met-CC vs LOD/LOD-CC comparison using the fixed-effect model: **(A)** effectiveness; and **(B)** safety. Notes: Met, metformin, CC, clomiphene citrate, LOD, laparoscopic ovarian drilling.

Effectiveness did not differ significantly between Met/Met-CC and LOD/LOD-CC in terms of live birth/ongoing pregnancy (RR = 1.02, 95% confidence interval [CI]: 0.87–1.21, z = 0.28; *p* = 0.780), pregnancy (RR = 0.98, 95% CI: 0.85–1.12, z = 0.29; *p* = 0.771), or rate of ovulation induction (RR = 0.99, 95% CI: 0.89–1.10, z = 0.16; *p* = 0.873; (Figure 3A).

Safety did not differ significantly between Met/Met-CC and LOD/LOD-CC in terms of miscarriage (RR = 0.79, 95% CI: 0.46–1.36, z = 0.86, *p* = 0.390), multiple pregnancy rates (RR = 9.39, 95% CI: 0.51–172.77, z = 1.51; *p* = 0.132). OHSS was rarely reported (Figure 3B). No data about ectopic pregnancy were provided in any of the five RCTs.

### Heterogeneity

The *I* ^2^ test results revealed moderate heterogeneity with regards to live birth/ongoing pregnancy (*I* ^2^ = 51.4%; *p* = 0.083) and rate of ovulation induction (*I* ^2^ = 50.9%; *p* < 0.086), but low heterogeneity regarding pregnancy rate (*I* ^2^ = 44.6%; *p* = 0.125; Figure 3A).

The *I* ^2^ test result revealed no heterogeneity with regards to miscarriage (*I* ^2^ = 0.0%; *p* = 0.952). A multiple pregnancy rate of 2.9% (4/138 women) was reported in the Met-CC group of one study (Abu Hashim et al., 2011) (Figure 3B).

### Sensitivity Analysis

Pooling based on a random-effects model (M-H heterogeneity) resulted in similar live-birth/ongoing pregnancy rates (RR = 1.04, 95% CI: 0.76–1.42, z = 0.25, *p* = 0.805; *I* ^2^ = 51.4%, Tau^2^ = 0.059, *p* = 0.083), pregnancy (RR = 1.00, 95% CI: 0.80–1.25, z = 0.02, *p* = 0.984; *I* ^2^ = 44.6%, Tau^2^ = 0.027, *p* = 0.125), and rate of ovulation induction (RR = 1.02, 95% CI 0.86–1.21, *z* = 0.28, *p* = 0.776; *I* ^2^ = 50.9%, Tau^2^ = 0.018, *p* = 0.086; (Supplementary Appendix S3, Figure 1A). Sensitivity analysis using the leave-one-out method revealed that the results when omitting each study were similar to those obtained when all studies were included (Supplementary Appendix S3, Figure 1B). As such, sensitivity analysis indicated that the results were stable.

### Meta-Regression Analysis to Explore Sources of Heterogeneity

We assessed the treatment methods, follow-up period after pregnancy, subjects’ country of residence, and samples using logistic meta-regression analysis. The results implied that different follow-up periods contributed to 15.43% of the heterogeneity (*I* ^2^ residual = 59.84%, Tau^2^ = 0.073; *p* = 0.271), and that the different countries of the participants accounted for 26.17% of the heterogeneity (*I* ^2^ residual = 40.43%, Tau^2^ = 0.064; *p* = 0.245). The adjusted *R* ^2^ values of the regression results were negative for both treatment used and sample size since the number of included studies was small (Supplementary Appendix S4).

### Subgroup Analysis and Investigation of Heterogeneity

The included studies were stratified by treatment methods. No significant difference occurred between Met-CC and LOD (RR = 0.97, 95% CI: 0.81–1.17, *z* = 0.27, *p* = 0.785; *I* ^2^ = 0.0%, *p* = 0.834) or between Met and LOD (RR = 1.11, 95% CI: 0.77–1.59, *z* = 0.56, *p* = 0.576; *I* ^2^ = 85.5%, *p* = 0.009). Only one RCT analyzing Met-CC vs LOD-CC was included in the present meta-analysis (Supplementary Appendix S5, Figure 2A).

The included studies were then divided into subgroups according to follow-up period. After excluding the study by Hamed et al., in which the pregnancy was only followed-up until 13 weeks’ gestation to obtain first trimester abortion and ongoing pregnancy data, no significant effect was found (RR = 1.09, 95% CI: 0.93–1.29, *z* = 1.04; *p* = 0.296), and there was low RR variation due to heterogeneity (*I* ^2^ = 38.4%; *p* = 0.182) in the live birth subgroup (Supplementary Appendix S5, Figure 2B).

Next, the participants were divided into subgroups based on the participants’ countries. No significant difference occurred between any of the treatment methods in either Egypt (RR = 0.91, 95% CI: 0.75–1.10, *z* = 1.00, *p* = 0.316; *I* ^2^ = 64.7%, *p* = 0.092) or Italy (RR = 1.34, 95% CI: 0.98–1.85, *z* = 1.83, *p* = 0.068; *I* ^2^ = 17.0%, *p* = 0.300; Supplementary Appendix S5, Figure 2C).

### Meta-Analysis Was Reperformed After Excluding a Study With a Concern Note by the Journal

The RCT published by Hashim HA (2011), which included the largest number of patients and cited by two reviews (Yu et al., 2017; Bordewijk et al., 2020a) and Guidelines (Teede et al., 2018) are essential and had recently been suspected by rationalization on the study and data integrity (Bordewijk et al., 2020b; Bordewijk et al., 2020c; Bordewijk et al., 2020d). Although Hashim HA. has explained the data and opposed undue stigmatization (Abu Hashim, 2020) a meta-analysis was reperformed to be rigorous after excluding Hashim HA (2011) before a final decision by the journal to retract the paper or concern note. (Supplementary Appendix S6).

### Assessment of Reporting Bias

**FIGURE 4:**
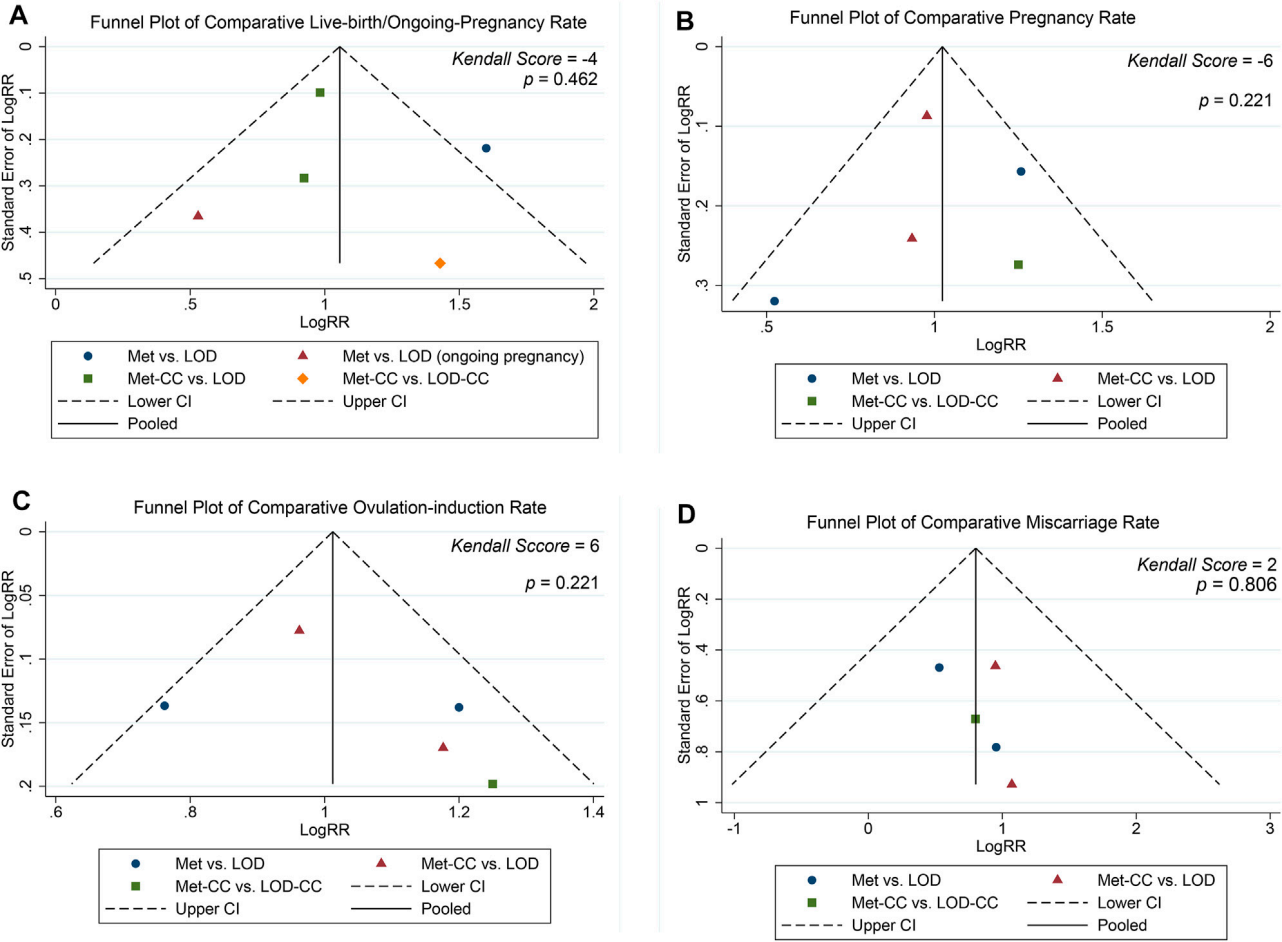
Funnel plot of **(A)** live birth/ongoing pregnancy, **(B)** pregnancy **(C)** ovulation induction, and **(D)** miscarriage rates.

The funnel plot in Figure 4 shows roughly symmetrical distributions when fertility outcome was compared among the five included RCTs. No significant evidence of publication bias was found in the present meta-analysis using Egger linear regression (*p* = 0.170, 0.291, 0.514, and 0.908 for live birth/ongoing pregnancy, pregnancy, ovulation induction, and miscarriage rates, respectively) or the Begg rank correlation test (Kendall score, *z* and *p* value with continuity correction = −4, −0.73, and 0.462 in live birth/ongoing pregnancy; −6, 1.22, and 0.221 in pregnancy; 6, 1.22, and 0.221 in ovulation induction; and 2, 0.24, 0.806 in miscarriage rates, respectively; Table 2).

**TABLE 2:** Egger linear regression and Begg rank correlation.

Fertility	Egger linear Regression				Begg rank correlation			
	Coef	95% CI	t	*p*	Kendall score (P-Q)	SD^#^	*z* ^*^	*p* ^*^
Ovulation-induction rate	1.322	−4.373–7.017	0.74	0.514	6	4.08	1.22	0.221
Pregnancy rate	−6.109	−21.33–9.111	−1.28	0.291	−6	4.08	1.22	0.221
Live-birth/ongoing pregnancy rate	−8.233	−22.78–6.317	−1.80	0.170	−4	4.08	0.73	0.462
Miscarriage rate	6.962	−168.7–182.7	0.13	0.908	2	4.08	0.24	0.806

Note: ^#^, SD, standard deviation of score; *, *z* and *p* values were continuity corrected. ^*^, Z and *p* values were continuity corrected.

### Comparison of Other Safety Outcomes and Costs

Although drug-related AEs occurred at rates between 5.5% and 22.2%; the symptoms were self-limiting and did not affect treatment continuation. LOD was completed successfully without any intra- or post-operative complications in all but three patients: one with obesity who failed the laparoscopy and two who showed minimal endometriosis and avascular pelvic adhesions (Palomba et al., 2010). Costs were reported in one included trial (€50.16 for Met vs €1050 for LOD; Table 1). (Palomba et al., 2004)

### Quality of the Body of Evidence

We found no evidence of a difference between Met/Met-CC and LOD, as well as between Met-CC and LOD-CC in terms of live birth/ongoing pregnancy, pregnancy, ovulation induction, and miscarriage rates. The “summary of findings” table showed that the quality of evidence was moderate in the Met-CC vs. LOD comparison, low for the pregnancy and ovulation induction rates of the Met vs. LOD comparison, and very low for the Met-CC vs. LOD-CC comparison, live birth/ongoing pregnancy, and miscarriage rates of the Met vs. LOD comparison (Supplementary Appendix S7, Table 1).

## Comments

### Summary of Main Findings

In patients with anovulatory, infertility-related CCR-PCOS, our main analysis including all studies indicated no significant difference in effectiveness or safety between Met/Met-CC and LOD, as well as between Met-CC and LOD-CC. The robustness of this result was evaluated using sensitivity analysis. The quality of evidence was very low to moderate.

Moderate-quality evidence suggested that Met-CC and LOD should be recommended as parallel treatments for CCR-PCOS. The live-birth rates associated with Met-CC and LOD were 57.06% and 58.58%, respectively. The miscarriage rate was similar between the two (6.13% vs. 6.51%). The *I* ^2^ tests results revealed no heterogeneity. With regards to costs, Met-CC is slightly superior to LOD. These findings confirmed the latest international guideline that Met-CC and LOD should be recommended as second-line treatments, with no definitive treatment timeline specified (NHRMC, 2018; Teede et al., 2018).

Very-low quality evidence indicated that there was uncertainty about the effectiveness and safety of Met and LOD in terms of live birth/ongoing pregnancy and miscarriage rates. Similarly, the effectiveness and safety of Met-CC and LOD-CC were unclear in terms of live birth, pregnancy, ovulation induction, and miscarriage rates. *I* ^2^ tests results indicated high heterogeneity in live birth/ongoing pregnancy rate between Met and LOD. Low-quality evidence indicated no difference in pregnancy or ovulation induction rates between Met and LOD. Our results showed that Met is a promising drug and were consistent with the international evidence-based guideline, which recommend Met alone to manage PCOS.

### Comparison With Existing Literature

The results of Yu et al. (2017) showed no difference in live birth, pregnancy, ovulation induction, or miscarriage rates between Met/Met-CC and LOD (Yu et al., 2017). The same authors concluded that no recommendation could be made regarding ovulation induction in patients with CCR-PCOS because the quality of evidence was low and the confidence intervals wide. The present meta-analysis showed similar results, with some differences. For example, we included more RCTs comparing Met-CC with LOD-CC. Moreover, Yu et al. compared pregnancy per cycle, abortion rate per pregnancy, and multiple pregnancy rate per pregnancy as their outcomes. Yu et al. also classified the 2011 study by Hashim et al. as having high risk of bias because it failed to blind outcome assessment, failing to consider that all outcomes were objective indicators. As such, the evidence quality was assessed differently between the study by Yu et al. and the present study.

Bordewijk et al. (2020) concluded that LOD/LOD-CC medical ovulation induction may lead to lower live birth rates in women with anovulatory CCR-PCOS than medical ovulation induction alone (Bordewijk et al., 2020a). This conclusion was inconsistent with the present meta-analysis. Bordewijk et al. included three non-RCTs (Malkawi et al., 2003; Ashrafinia et al., 2009; Elgafor el sharkwy, 2013) and three RCTs (Palomba et al., 2004; Palomba et al., 2010; Abu Hashim et al., 2011) to compare the effectiveness of Met/Met-CC with that of LOD. Furthermore, we concluded that the 2004 study by Palomba et al. should be classified as a comparison of Met with LOD rather than of Met-CC with LOD, because CC was only given to women who failed to achieve ovulation after the trial in that study (Palomba et al., 2004).

### Strengths and Limitations

The strengths of the present systematic review included the extensive search strategy, as well as the meta-regression, subgroup, and sensitivity analyses. All included studies were RCTs with no publication bias, and the robustness of their results was confirmed by sensitivity analysis.

However, the present meta-analysis also had several limitations. For example, the number of included RCTs and participants was limited. In addition, the treatment methods were potential source of heterogeneity. The two RCTs comparing Met with LOD were highly heterogeneous, while the one comparing Met-CC with LOD-CC had a small sample size. No RCT compared Met with LOD-CC. Because the quality of evidence was low or very low, no conclusion could be drawn regarding the comparison of Met with LOD or of Met-CC with LOD-CC. Lastly, variation in the follow-up periods after pregnancy may have impacted fertility results. That said, the two groups in the trials were subject to the same experimental conditions in terms of follow-up time, which may have reduced heterogeneity.

## Conclusion and Implications

Although moderate-quality evidence suggested that Met-CC and LOD should be recommended as parallel, second-line therapies for patients with CCR-PCOS, the present study indicates that Met-CC should be recommended as the optimum treatment for patients with CCR-PCOS because it is cheap, safe, and different from LOD, whose effect depends on operator proficiency; LOD intervention might be the first choice for patients with CCR-PCOS if they are willing to undergo diagnostic laparoscopy.

Very low-to low-quality evidence has suggested that there is little or no difference in effectiveness or safety between Met and LOD, as well as between Met-CC and Met-CC in women with CCR-PCOS, but our results showed that Met is a promising drug that could play a more important role in CCR-PCOS treatment because it is effective, safe, and cheap. Specifically, Met is an old drug with new applications, including PCOS treatment (Wang et al., 2017; Zhou et al., 2018; Sharpe et al., 2019) and Met treatment during pregnancy does not influence metabolic profile in women with PCOS (Underdal et al., 2018). Furthermore, although a recently published follow-up study of two RCTs suggested that *in utero* exposure to Met increases the risk of overweightness in early childhood (Greenhill, 2018; Hanem et al., 2018) there is fair evidence that Met alone does not increase rates of miscarriage when stopped at the initiation of pregnancy (Practice Committee of the American Society for Reproductive Medicine, 2017).

LOD reduces both testosterone and the luteinizing hormone/follicle-stimulating hormone ratio in women with PCOS, and it improves clinical outcome (Sinha et al., 2019). Currently, LOD can be performed using monopolar, bipolar, or laser diathermy; even ultrasound-guided transvaginal ovarian needle drilling has been used to induce ovulation in anovulatory PCOS (Farquhar et al., 2007; Rezk et al., 2016; Kandil et al., 2018). With developing technology, LOD has emerged as an ideal alternative to letrozole, maximizing ovulation induction and pregnancy benefits without the problems associated with ovarian wedge resection (Abu Hashim et al., 2013; Zahiri Sorouri et al., 2015; El-Sayed et al., 2017; Debras et al., 2019; Yu et al., 2019; Zhang et al., 2019). Currently, the latest international evidence-based guideline recommended letrozole as first line pharmacological treatment for anovulation women with PCOS to improve ovulation, pregnancy and live-birth (NHRMC, 2018). The results of present meta-analysis did not found difference in effectiveness or safety between Met/Met-CC and LOD/LOD-CC, and thus we could safely deduce that Met is a promising drug.

Additional large RCTs with adequate blinding are needed to more precisely estimate the difference between Met/Met-CC and LOD/LOD-CC. Such trials should comprehensively evaluate outcomes, including live birth, pregnancy, ovulation induction, AEs (multiple pregnancy, miscarriage, ectopic pregnancy, OHSS, drug-related AEs, and surgical complications), costs, patient satisfaction, long-term benefits (spontaneous resumption of ovulation and menstruation), as well as the potential risks of LOD (such as premature ovarian failure).

In conclusion, there is no evidence on the difference in the outcomes between the two interventions regarding ovulation, pregnancy, and live birth. As LOD is an invasive procedure and carries inherent risks, the use of metformin with or without clomiphene should be the second-line treatment for women with polycystic ovary syndrome (PCOS) who do not ovulate only by clomiphene citrate.

## Data Availability

The datasets presented in this study can be found in online repositories. The names of the repository/repositories and accession number(s) can be found in the article/Supplementary Material.
