# Supplementary material for: Metformin With or Without Clomiphene Citrate Versus Laparoscopic Ovarian Drilling With or Without Clomiphene Citrate to Treat Patients With Clomiphene Citrate-Resistant Polycystic Ovary Syndrome: A Systematic Review and Meta-Analysis
Source: Front Pharmacol. 2022 Jun 22;13:576458. doi: 10.3389/fphar.2022.576458 (PMC9256960; doi:10.3389/fphar.2022.576458)
Supplement: Supplementary file 1 [file DataSheet2.PDF]

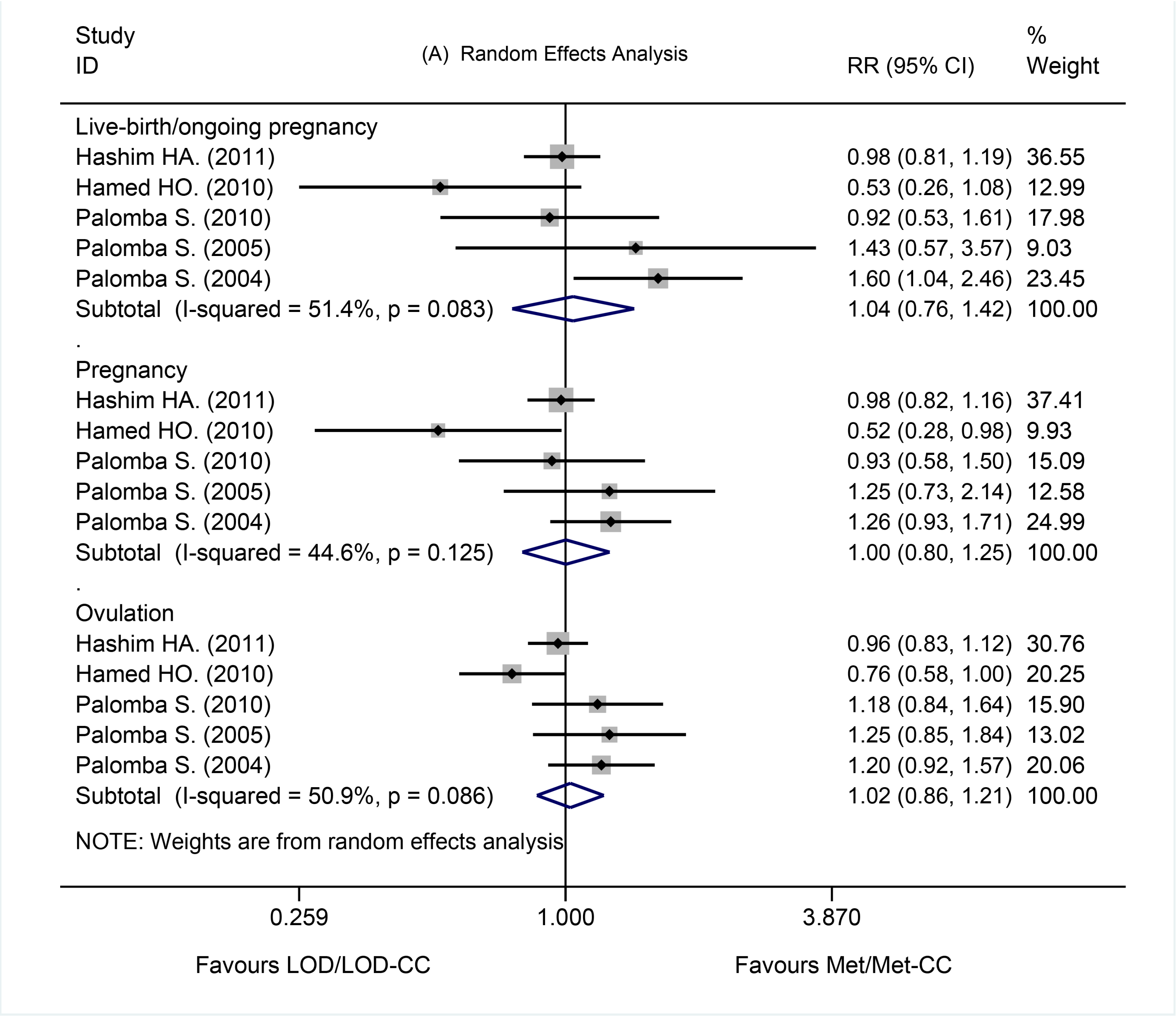
Study
ID
(A) Random Effects Analysis
RR (95% CI)
%
Weight
Live-birth/ongoing pregnancy
Hashim HA. (2011) 0.98 (0.81, 1.19) 36.55
Hamed HO. (2010) 0.53 (0.26, 1.08) 12.99
Palomba S. (2010) 0.92 (0.53, 1.61) 17.98
Palomba S. (2005) 1.43 (0.57, 3.57) 9.03
Palomba S. (2004) 1.60 (1.04, 2.46) 23.45
Subtotal (I-squared = 51.4%, p = 0.083) 1.04 (0.76, 1.42) 100.00
.
Pregnancy
Hashim HA. (2011) 0.98 (0.82, 1.16) 37.41
Hamed HO. (2010) 0.52 (0.28, 0.98) 9.93
Palomba S. (2010) 0.93 (0.58, 1.50) 15.09
Palomba S. (2005) 1.25 (0.73, 2.14) 12.58
Palomba S. (2004) 1.26 (0.93, 1.71) 24.99
Subtotal (I-squared = 44.6%, p = 0.125) 1.00 (0.80, 1.25) 100.00
.
Ovulation
Hashim HA. (2011) 0.96 (0.83, 1.12) 30.76
Hamed HO. (2010) 0.76 (0.58, 1.00) 20.25
Palomba S. (2010) 1.18 (0.84, 1.64) 15.90
Palomba S. (2005) 1.25 (0.85, 1.84) 13.02
Palomba S. (2004) 1.20 (0.92, 1.57) 20.06
Subtotal (I-squared = 50.9%, p = 0.086) 1.02 (0.86, 1.21) 100.00
NOTE: Weights are from random effects analysis
0.259
1.000
3.870
Favours LOD/LOD-CC
Favours Met/Met-CC

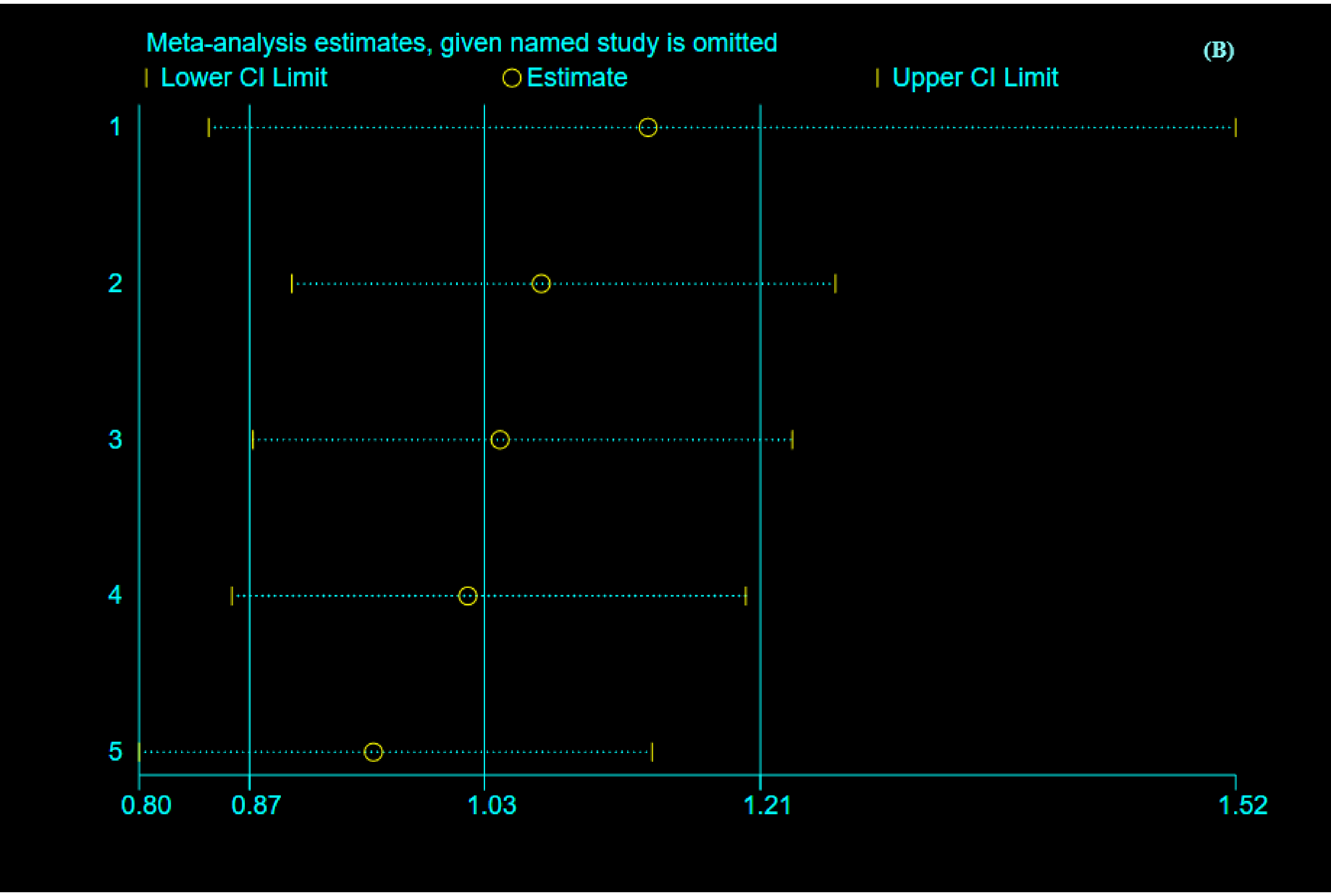
Meta-analysis estimates, given named study is omitted
(B)
Lower CI Limit
Estimate
Upper CI Limit
1
2
3
4
5
0.80
0.87
1.03
1.21
1.52

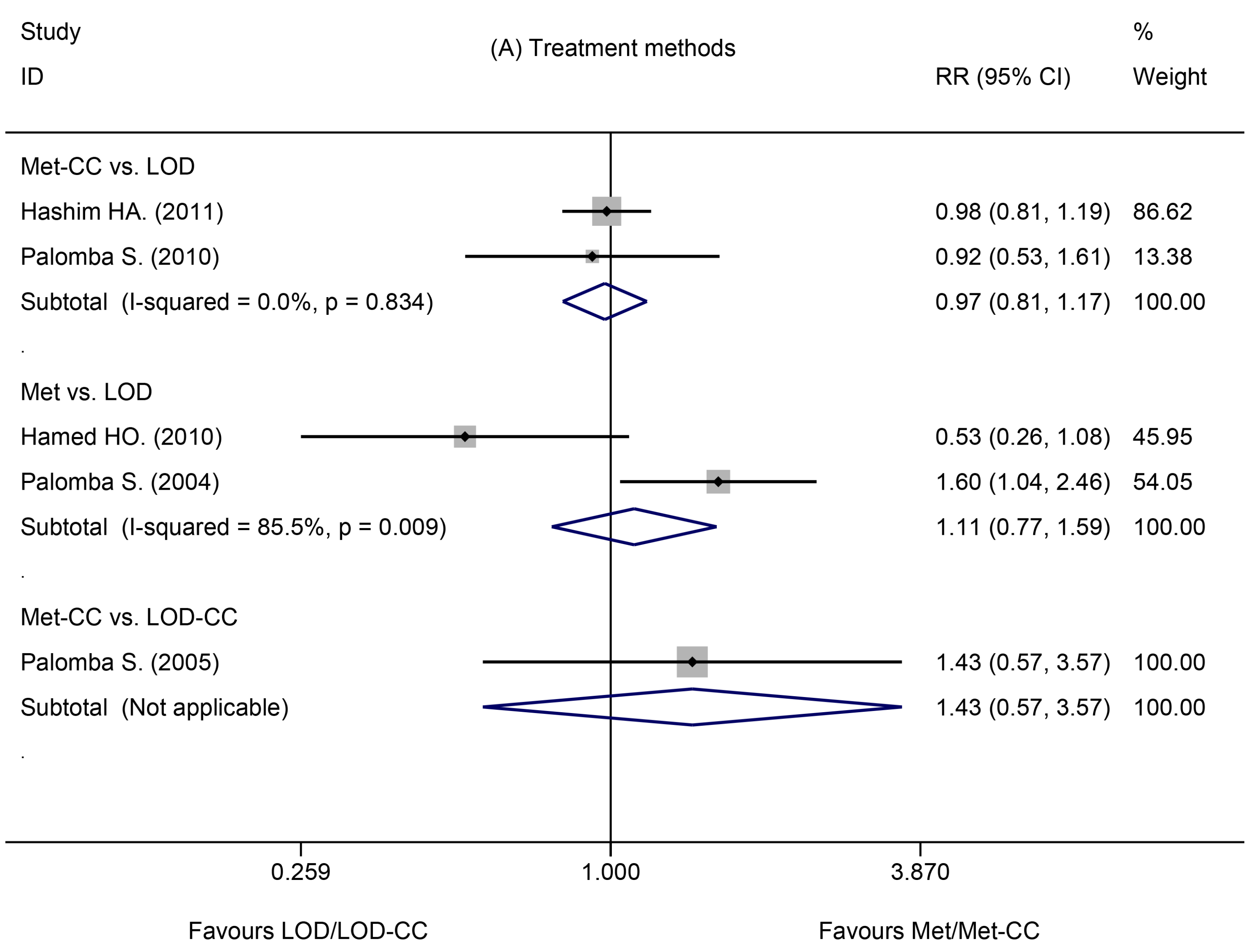

Study
ID
(A) Treatment methods
RR (95% CI)
%
Weight
Met-CC vs. LOD
Hashim HA. (2011) 0.98 (0.81, 1.19) 86.62
Palomba S. (2010) 0.92 (0.53, 1.61) 13.38
Subtotal (I-squared = 0.0%, p = 0.834) 0.97 (0.81, 1.17) 100.00
.
Met vs. LOD
Hamed HO. (2010) 0.53 (0.26, 1.08) 45.95
Palomba S. (2004) 1.60 (1.04, 2.46) 54.05
Subtotal (I-squared = 85.5%, p = 0.009) 1.11 (0.77, 1.59) 100.00
.
Met-CC vs. LOD-CC
Palomba S. (2005) 1.43 (0.57, 3.57) 100.00
Subtotal (Not applicable) 1.43 (0.57, 3.57) 100.00
.
0.259
1.000
3.870
Favours LOD/LOD-CC
Favours Met/Met-CC

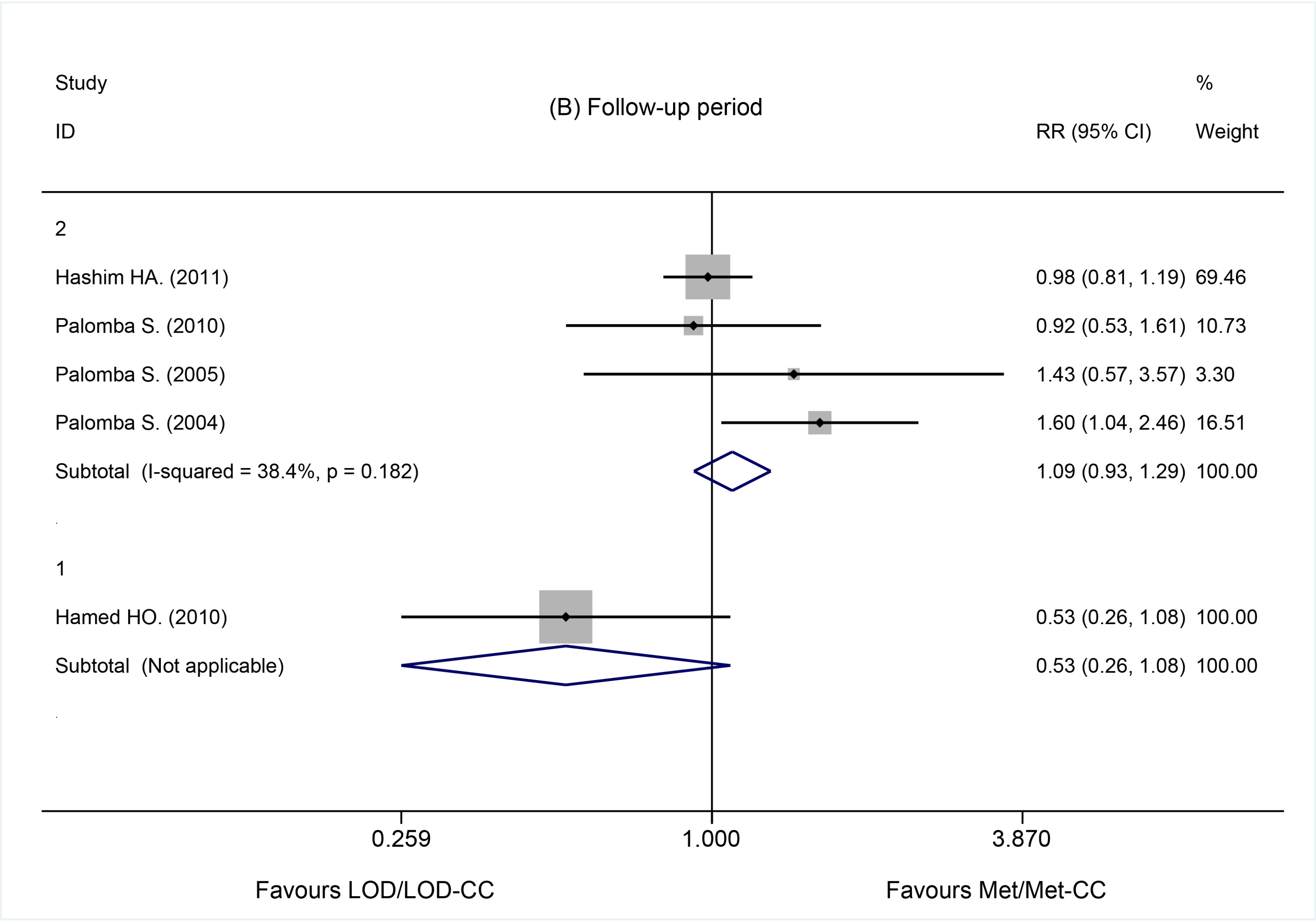
(B) Follow-up period
Study
ID
RR (95% CI)
%
Weight
2
Hashim HA. (2011) 0.98 (0.81, 1.19) 69.46
Palomba S. (2010) 0.92 (0.53, 1.61) 10.73
Palomba S. (2005) 1.43 (0.57, 3.57) 3.30
Palomba S. (2004) 1.60 (1.04, 2.46) 16.51
Subtotal (I-squared = 38.4%, p = 0.182) 1.09 (0.93, 1.29) 100.00
1
Hamed HO. (2010) 0.53 (0.26, 1.08) 100.00
Subtotal (Not applicable) 0.53 (0.26, 1.08) 100.00
0.259
1.000
3.870
Favours LOD/LOD-CC
Favours Met/Met-CC

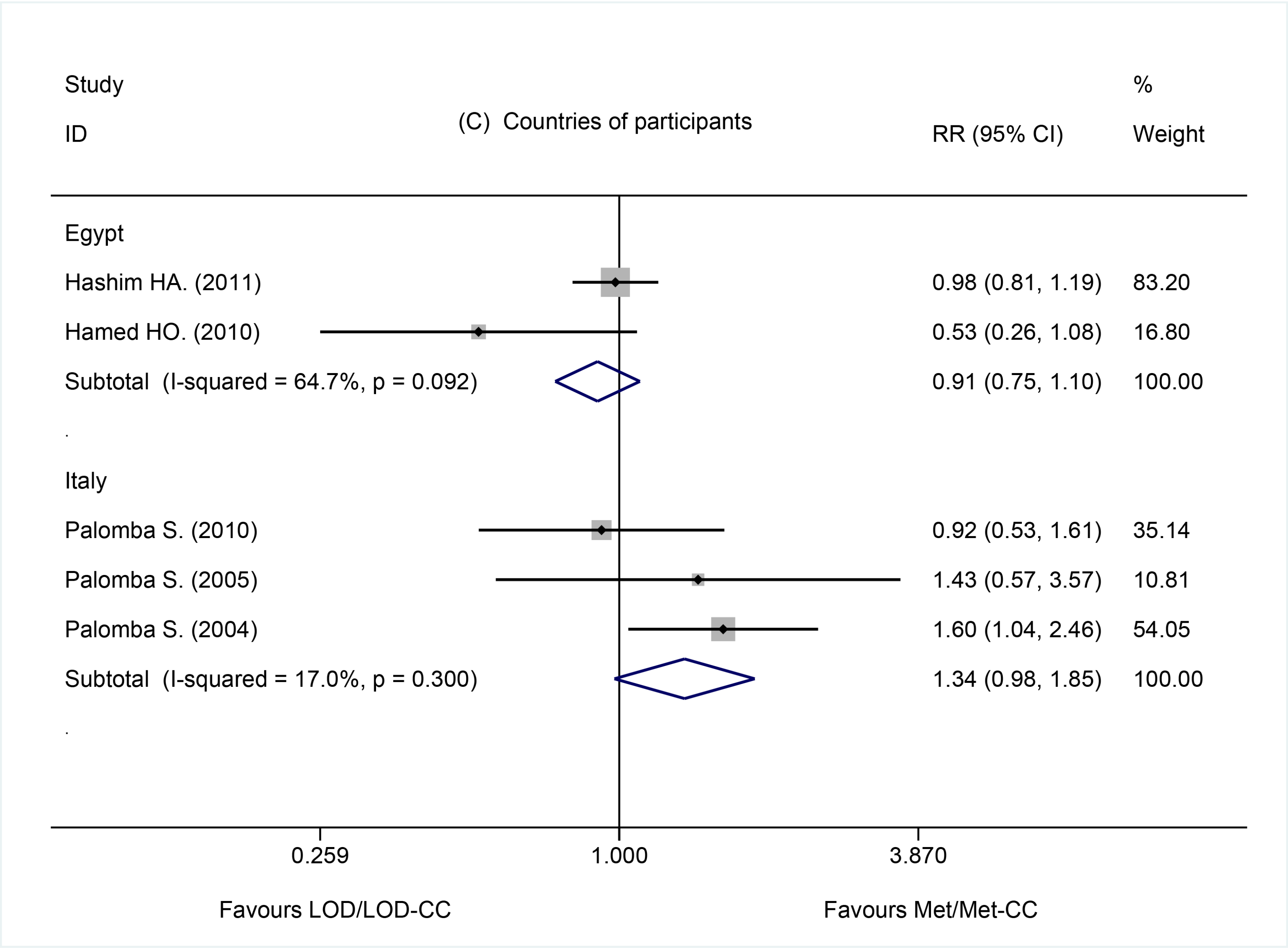
Study
ID
(C) Countries of participants
RR (95% CI)
%
Weight
Egypt
Hashim HA. (2011)
0.98 (0.81, 1.19)
83.20
Hamed HO. (2010)
0.53 (0.26, 1.08)
16.80
Subtotal (I-squared = 64.7%, p = 0.092)
0.91 (0.75, 1.10)
100.00
.
Italy
Palomba S. (2010)
0.92 (0.53, 1.61)
35.14
Palomba S. (2005)
1.43 (0.57, 3.57)
10.81
Palomba S. (2004)
1.60 (1.04, 2.46)
54.05
Subtotal (I-squared = 17.0%, p = 0.300)
1.34 (0.98, 1.85)
100.00
.
0.259
1.000
3.870
Favours LOD/LOD-CC
Favours Met/Met-CC

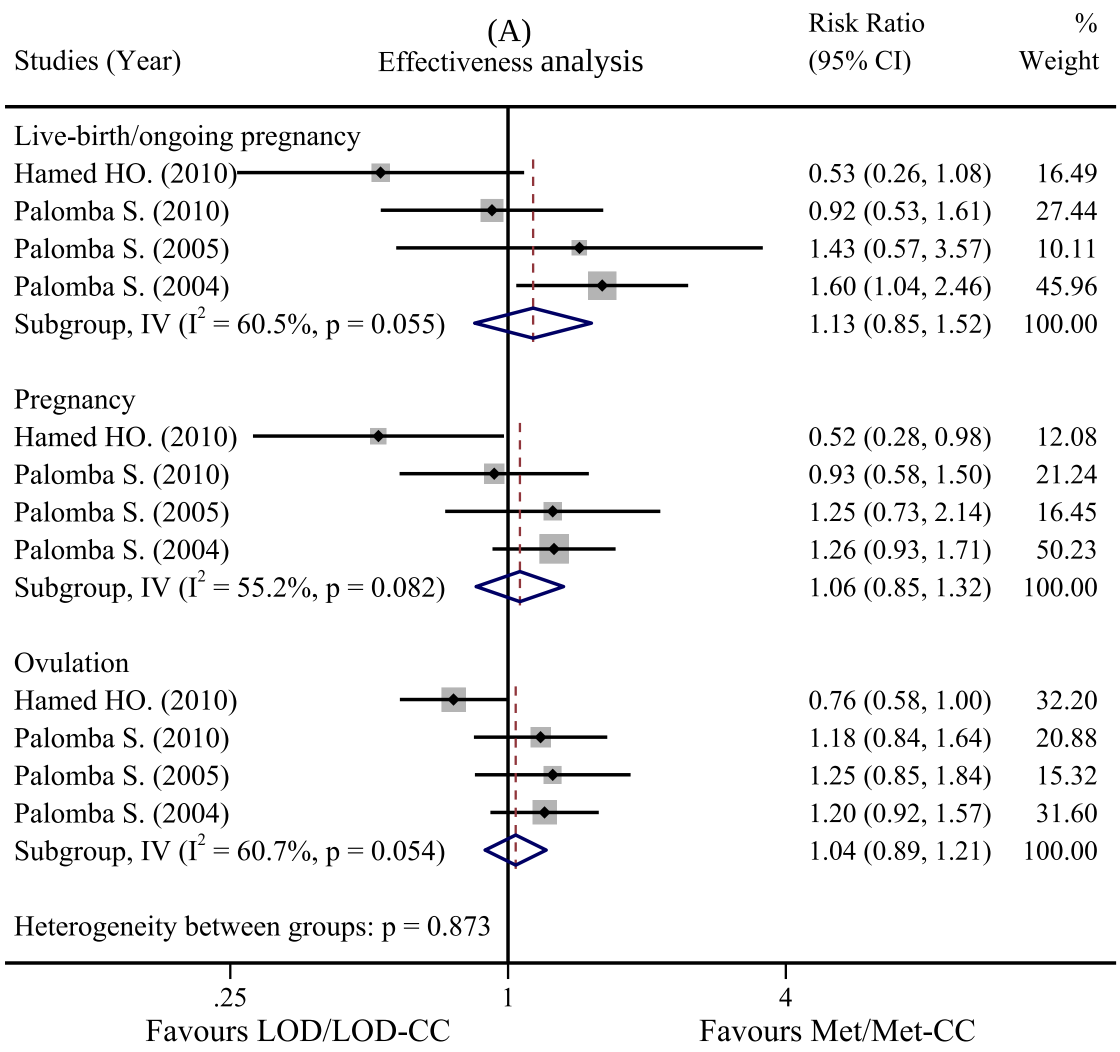
(A)
Effectiveness analysis
Studies (Year)
Risk Ratio (95% CI)
% Weight
Live-birth/ongoing pregnancy
Hamed HO. (2010) 0.53 (0.26, 1.08) 16.49
Palomba S. (2010) 0.92 (0.53, 1.61) 27.44
Palomba S. (2005) 1.43 (0.57, 3.57) 10.11
Palomba S. (2004) 1.60 (1.04, 2.46) 45.96
Subgroup, IV (I2 = 60.5%, p = 0.055) 1.13 (0.85, 1.52) 100.00
Pregnancy
Hamed HO. (2010) 0.52 (0.28, 0.98) 12.08
Palomba S. (2010) 0.93 (0.58, 1.50) 21.24
Palomba S. (2005) 1.25 (0.73, 2.14) 16.45
Palomba S. (2004) 1.26 (0.93, 1.71) 50.23
Subgroup, IV (I2 = 55.2%, p = 0.082) 1.06 (0.85, 1.32) 100.00
Ovulation
Hamed HO. (2010) 0.76 (0.58, 1.00) 32.20
Palomba S. (2010) 1.18 (0.84, 1.64) 20.88
Palomba S. (2005) 1.25 (0.85, 1.84) 15.32
Palomba S. (2004) 1.20 (0.92, 1.57) 31.60
Subgroup, IV (I2 = 60.7%, p = 0.054) 1.04 (0.89, 1.21) 100.00
Heterogeneity between groups: p = 0.873
.25
1
4
Favours LOD/LOD-CC
Favours Met/Met-CC

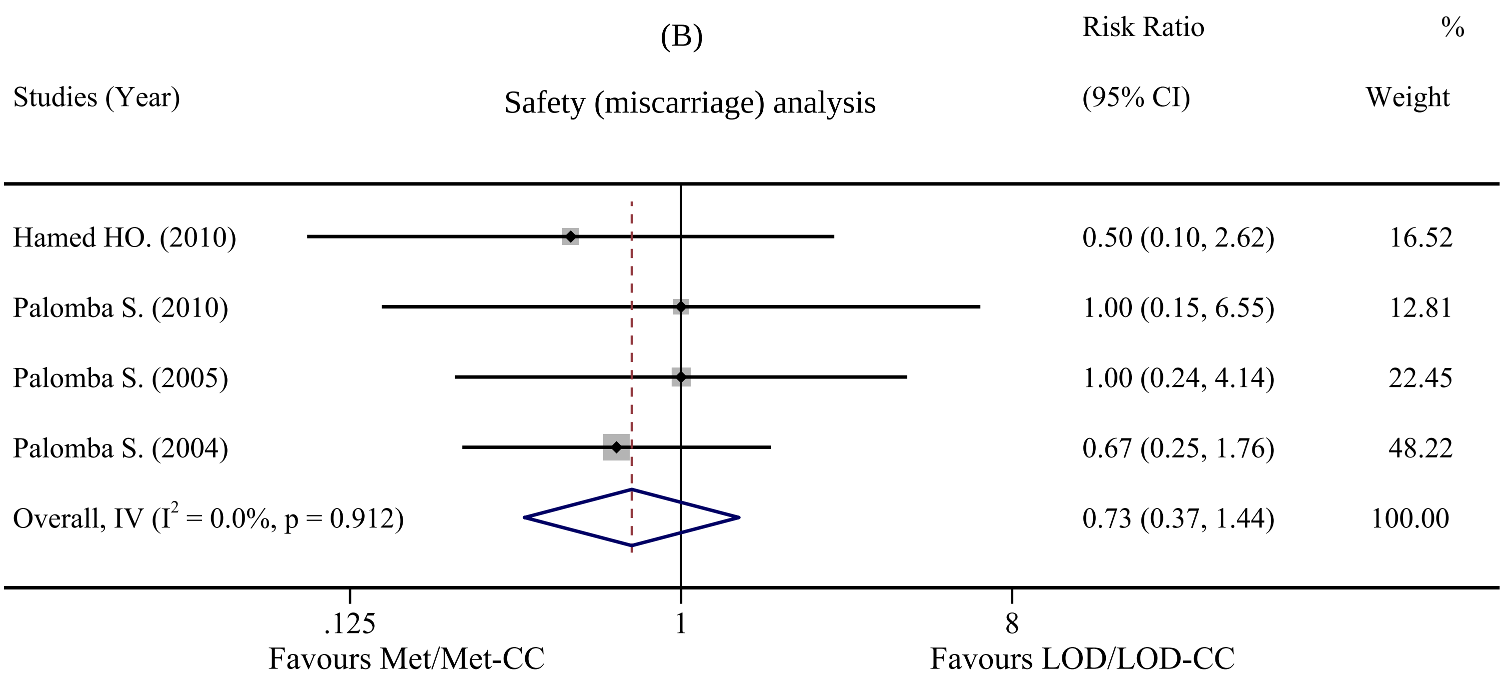
(B)
Safety (miscarriage) analysis
Studies (Year)
Risk Ratio
(95% CI)
%
Weight
Hamed HO. (2010)
0.50 (0.10, 2.62)
16.52
Palomba S. (2010)
1.00 (0.15, 6.55)
12.81
Palomba S. (2005)
1.00 (0.24, 4.14)
22.45
Palomba S. (2004)
0.67 (0.25, 1.76)
48.22
Overall, IV (I2 = 0.0%, p = 0.912)
0.73 (0.37, 1.44)
100.00
.125
1
8
Favours Met/Met-CC
Favours LOD/LOD-CC

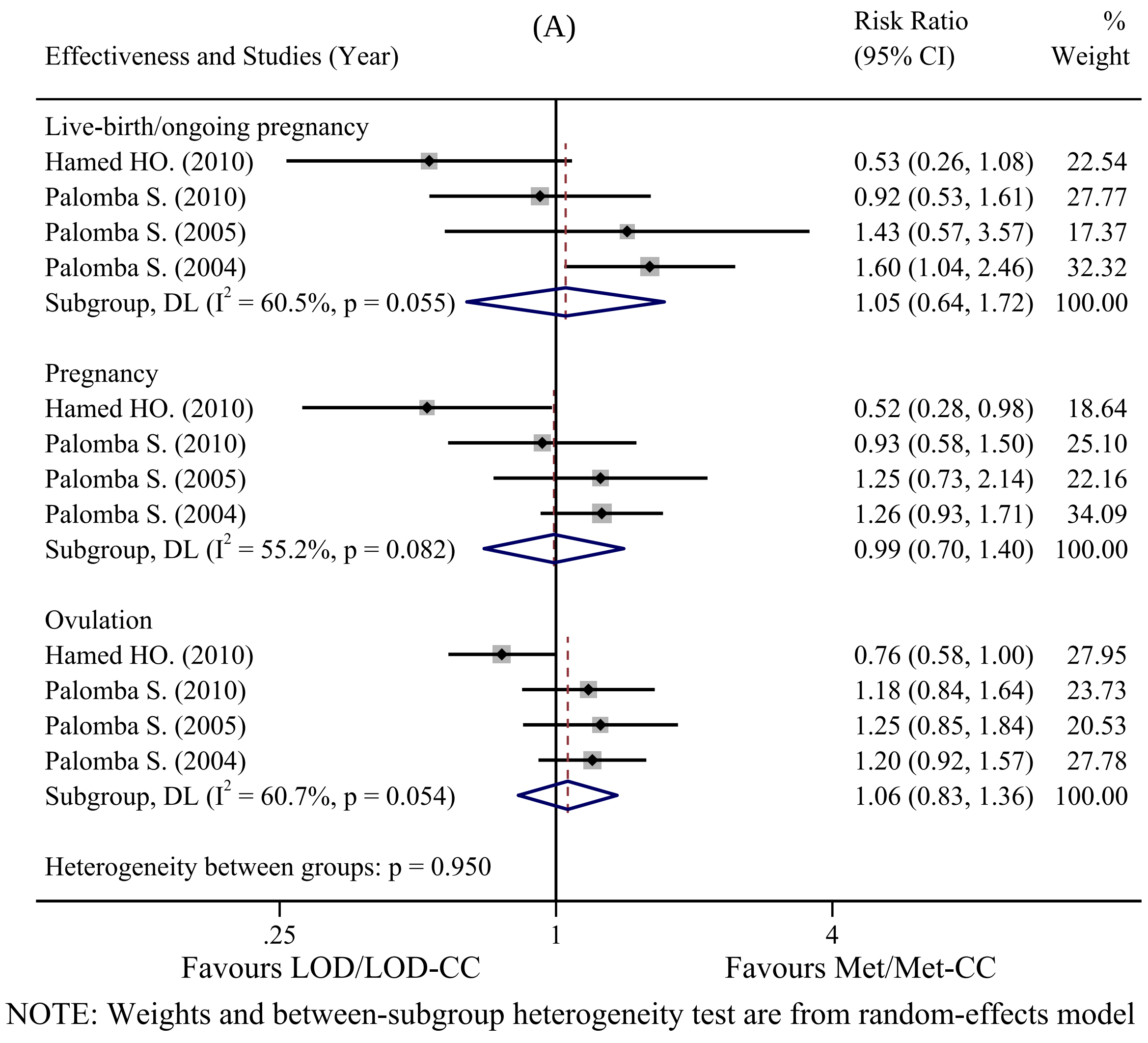
(A)
Effectiveness and Studies (Year)
Risk Ratio (95% CI)
% Weight
Live-birth/ongoing pregnancy
Hamed HO. (2010) 0.53 (0.26, 1.08) 22.54
Palomba S. (2010) 0.92 (0.53, 1.61) 27.77
Palomba S. (2005) 1.43 (0.57, 3.57) 17.37
Palomba S. (2004) 1.60 (1.04, 2.46) 32.32
Subgroup, DL (I2 = 60.5%, p = 0.055) 1.05 (0.64, 1.72) 100.00
Pregnancy
Hamed HO. (2010) 0.52 (0.28, 0.98) 18.64
Palomba S. (2010) 0.93 (0.58, 1.50) 25.10
Palomba S. (2005) 1.25 (0.73, 2.14) 22.16
Palomba S. (2004) 1.26 (0.93, 1.71) 34.09
Subgroup, DL (I2 = 55.2%, p = 0.082) 0.99 (0.70, 1.40) 100.00
Ovulation
Hamed HO. (2010) 0.76 (0.58, 1.00) 27.95
Palomba S. (2010) 1.18 (0.84, 1.64) 23.73
Palomba S. (2005) 1.25 (0.85, 1.84) 20.53
Palomba S. (2004) 1.20 (0.92, 1.57) 27.78
Subgroup, DL (I2 = 60.7%, p = 0.054) 1.06 (0.83, 1.36) 100.00
Heterogeneity between groups: p = 0.950
.25
1
4
Favours LOD/LOD-CC
Favours Met/Met-CC
NOTE: Weights and between-subgroup heterogeneity test are from random-effects model

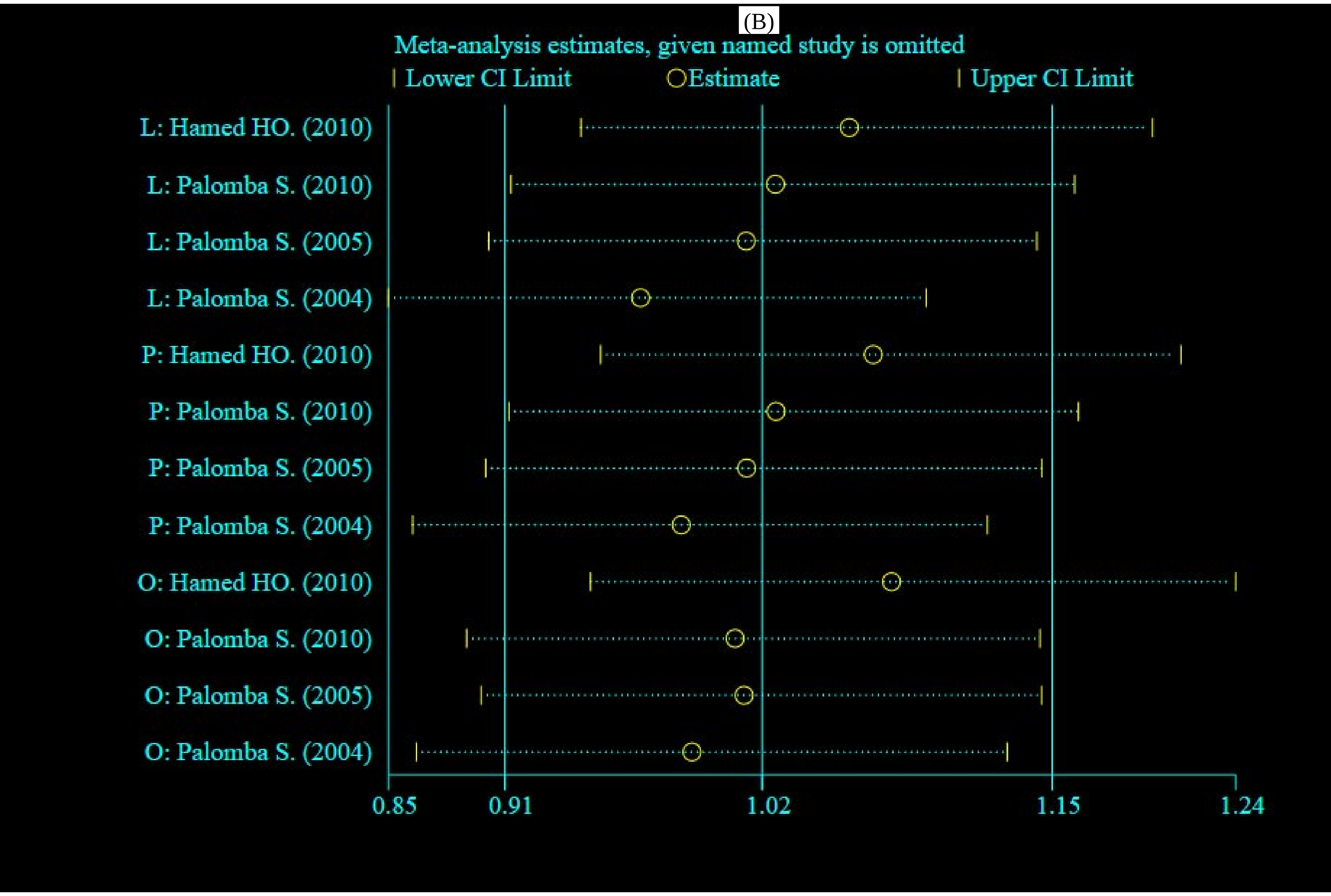
(B)
Meta-analysis estimates, given named study is omitted
Lower CI Limit
Estimate
Upper CI Limit
L: Hamed HO. (2010)
L: Palomba S. (2010)
L: Palomba S. (2005)
L: Palomba S. (2004)
P: Hamed HO. (2010)
P: Palomba S. (2010)
P: Palomba S. (2005)
P: Palomba S. (2004)
O: Hamed HO. (2010)
O: Palomba S. (2010)
O: Palomba S. (2005)
O: Palomba S. (2004)
0.85
0.91
1.02
1.15
1.24

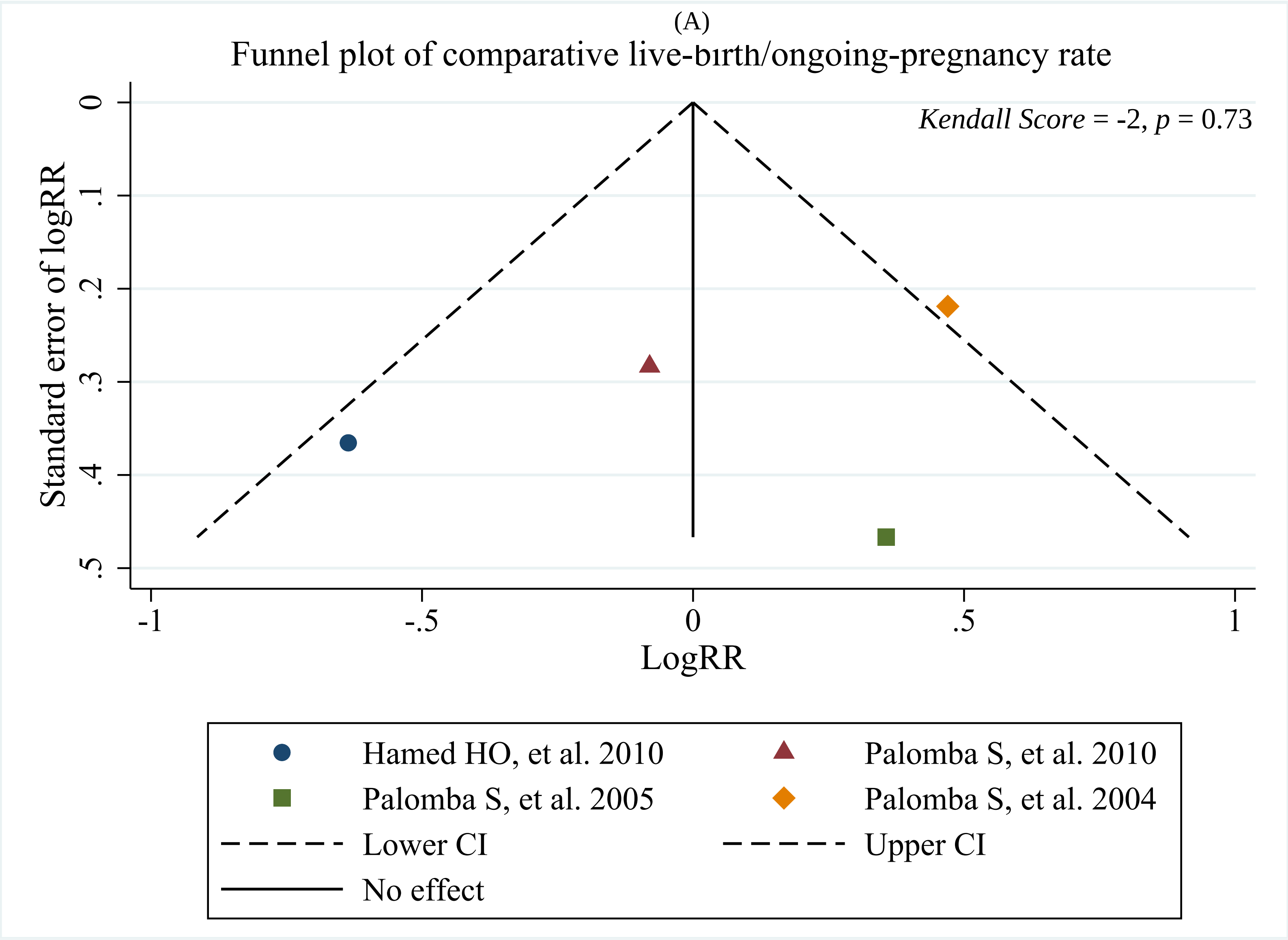
(A)
Funnel plot of comparative live-birth/ongoing-pregnancy rate
Kendall Score = -2, p = 0.73
Standard error of logRR
0
.1
.2
.3
.4
.5
-1
-.5
0
.5
1
LogRR
Hamed HO, et al. 2010
Palomba S, et al. 2010
Palomba S, et al. 2005
Palomba S, et al. 2004
Lower CI
Upper CI
No effect

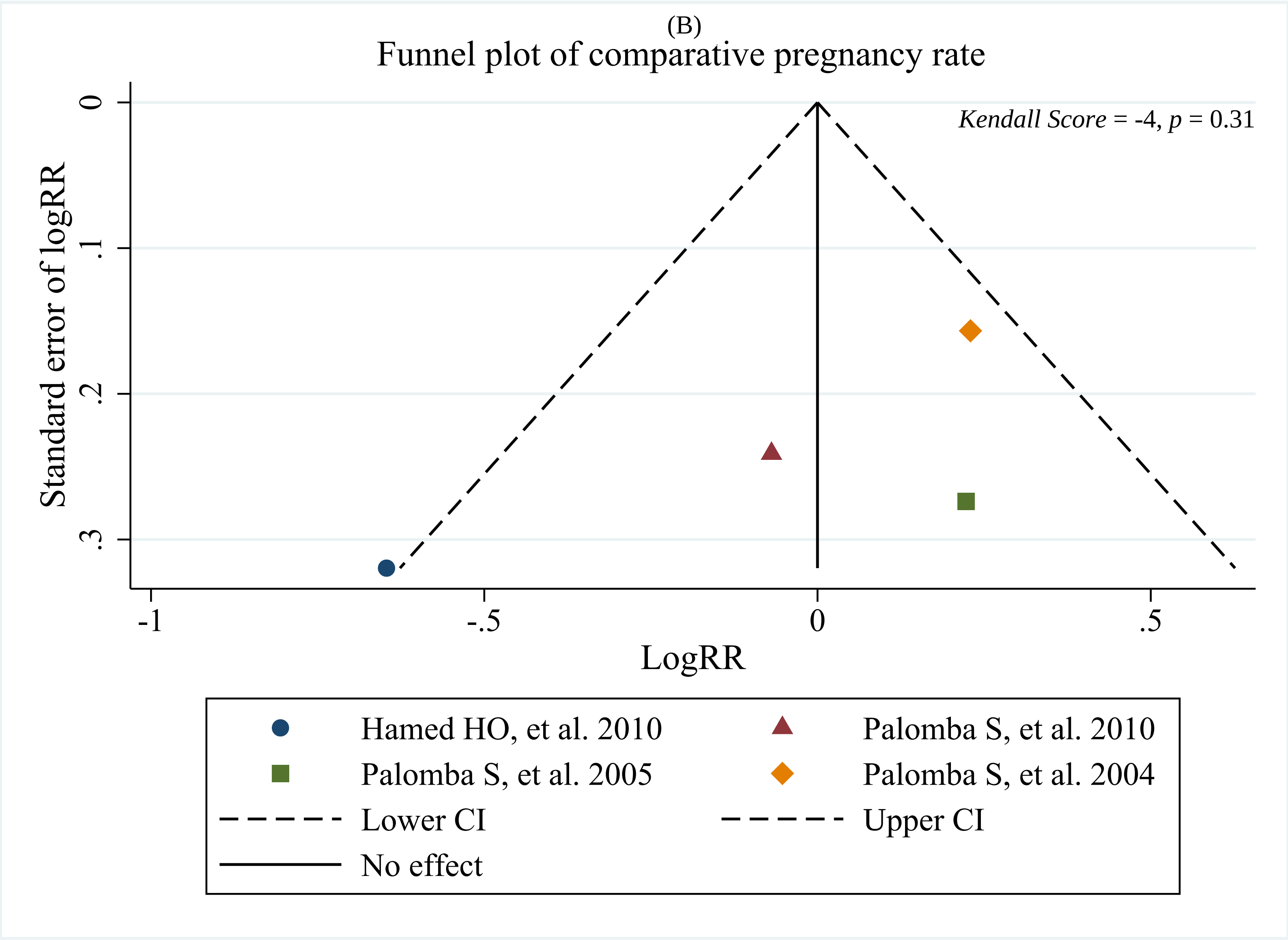
(B)
Funnel plot of comparative pregnancy rate
Kendall Score = -4, p = 0.31
Standard error of logRR
0
.1
.2
.3
-1
-.5
0
.5
LogRR
Hamed HO, et al. 2010
Palomba S, et al. 2010
Palomba S, et al. 2005
Palomba S, et al. 2004
Lower CI
Upper CI
No effect

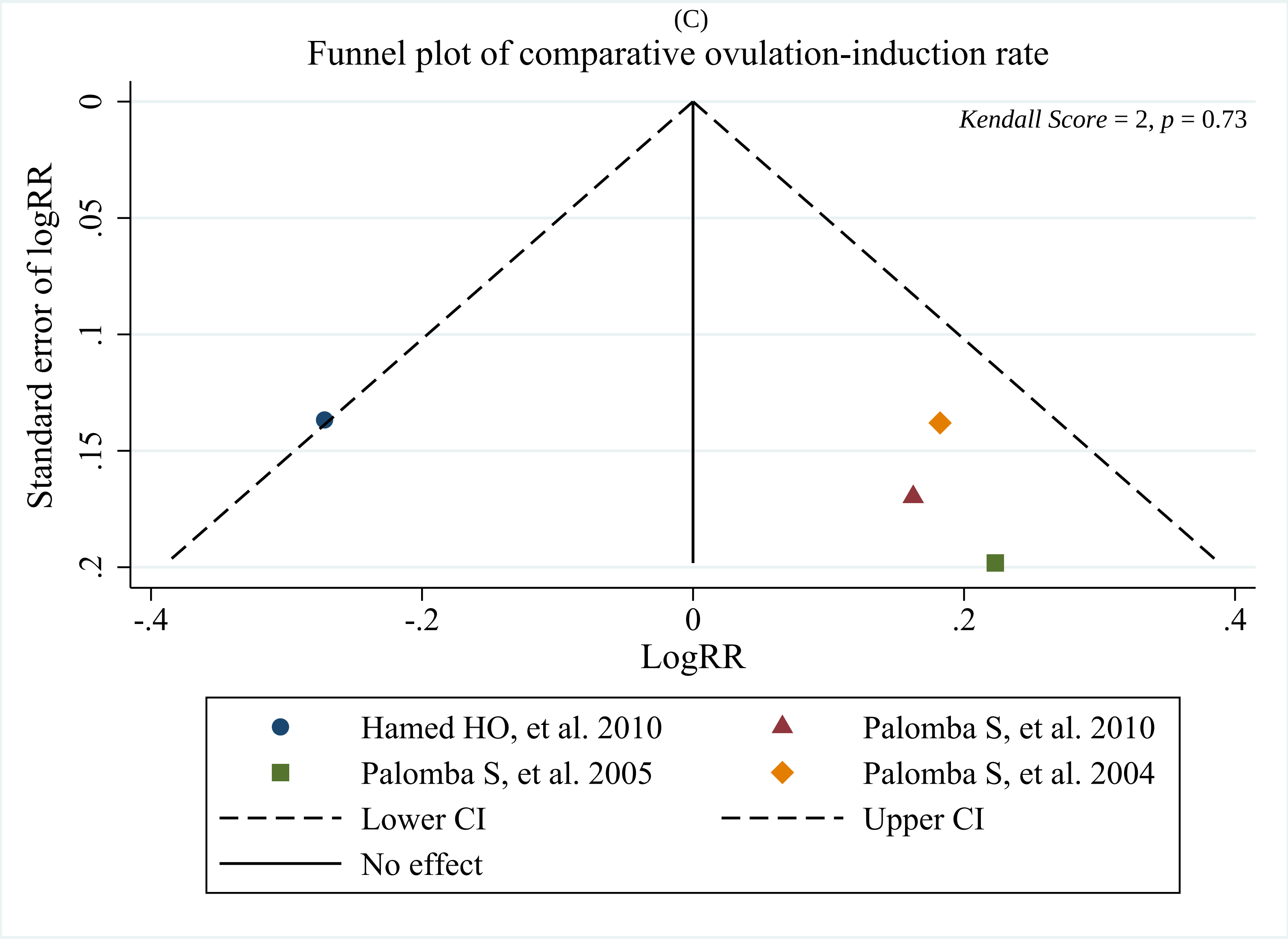
(C)
Funnel plot of comparative ovulation-induction rate
Kendall Score = 2, p = 0.73
Standard error of logRR
0
.05
.1
.15
.2
-.4
-.2
0
.2
.4
LogRR
Hamed HO, et al. 2010
Palomba S, et al. 2010
Palomba S, et al. 2005
Palomba S, et al. 2004
Lower CI
Upper CI
No effect

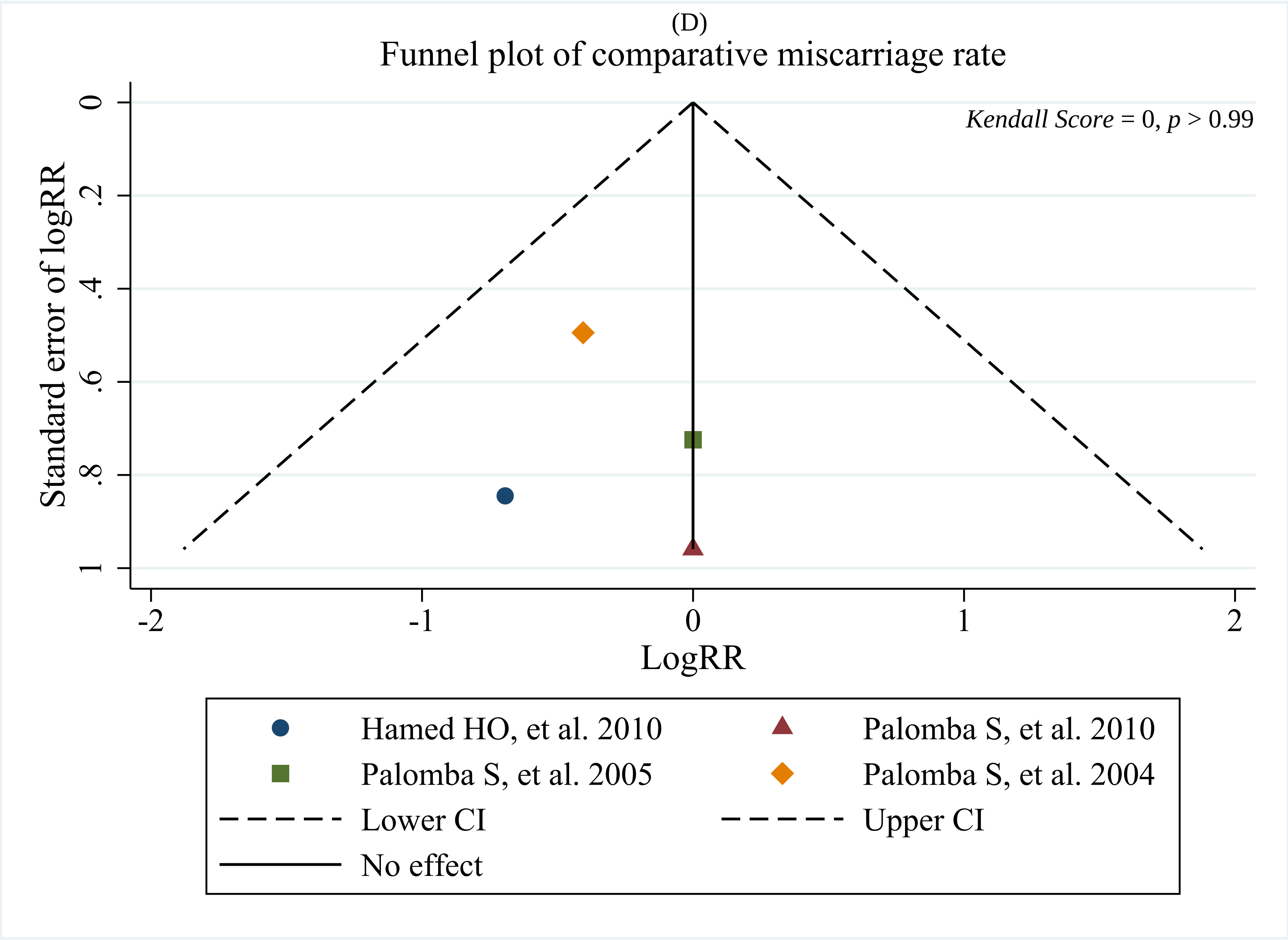
(D)
Funnel plot of comparative miscarriage rate
Kendall Score = 0, p > 0.99
Standard error of logRR
0
.2
.4
.6
.8
1
-2
-1
0
1
2
LogRR
Hamed HO, et al. 2010
Palomba S, et al. 2010
Palomba S, et al. 2005
Palomba S, et al. 2004
Lower CI
Upper CI
No effect

Met compared to LOD for CCR-PCOS

Patient or population: patients with CCR-PCOS
Settings: Inpatients
Intervention: Met
Comparison: LOD

Outcomes	Illustrative comparative risks* (95% CI) Assumed risk LOD	Corresponding risk Met	Relative effect (95% CI)	No of Participants (studies)	Quality of the evidence (GRADE)	Comments
**Live-birth/ongoing pregnancy** Live-birth/ultrasonography of uterus Follow-up: 6 months	**Study population**		**RR 1.11** (0.77 to 1.59)	230 (2 studies)	⊕⊖⊖⊖ **very low**[1,2,3]	
	**322 per 1000**	**357 per 1000** (248 to 512)				
	**Moderate**					
	**322 per 1000**	**357 per 1000** (248 to 512)				
**Miscarriage** Miscarriage Follow-up: 6 months	**Study population**		**RR 0.62** (0.27 to 1.42)	230 (2 studies)	⊕⊖⊖⊖ **very low**[1,2]	
	**113 per 1000**	**70 per 1000** (31 to 161)				
	**Moderate**					
	**113 per 1000**	**70 per 1000** (31 to 160)				
**Pregnancy** Serum β-HCG laboratory examination; ultrasonography of uterus Follow-up: 6 months	**Study population**		**RR 0.96** (0.73 to 1.27)	230 (2 studies)	⊕⊕⊖⊖ **low**[1,2]	
	**452 per 1000**	**434 per 1000** (330 to 574)				
	**Moderate**					
	**452 per 1000**	**434 per 1000** (330 to 574)				
**Ovulation-induction** The serum progesterone laboratory examination Follow-up: 6 months	**Study population**		**RR 0.96** (0.8 to 1.16)	230 (2 studies)	⊕⊕⊖⊖ **low**[1,2]	
	**670 per 1000**	**643 per 1000** (536 to 777)				
	**Moderate**					
	**670 per 1000**	**643 per 1000** (536 to 777)				

*The basis for the **assumed risk** (e.g. the median control group risk across studies) is provided in footnotes. The **corresponding risk** (and its 95% confidence interval) is based on the assumed risk in the comparison group and the **relative effect** of the intervention (and its 95% CI).

CI: Confidence interval; **RR:** Risk ratio;

GRADE Working Group grades of evidence
High quality: Further research is very unlikely to change our confidence in the estimate of effect.
Moderate quality: Further research is likely to have an important impact on our confidence in the estimate of effect and may change the estimate.
Low quality: Further research is very likely to have an important impact on our confidence in the estimate of effect and is likely to change the estimate.
Very low quality: We are very uncertain about the estimate.

[1] Downgraded one level for serious risk of performance bias.
[2] Downgraded one level for serious inconsistency.
[3] Downgraded two levels for very serious imprecision：the follow-up period after pregnancy was different

Met-CC compared to LOD for CCR-PCOS

Patient or population: patients with CCR-PCOS
Settings: Inpatients
Intervention: Met-CC
Comparison: LOD

Outcomes	Illustrative comparative risks* (95% CI) Assumed risk LOD	Corresponding risk Met-CC	Relative effect (95% CI)	No of Participants (studies)	Quality of the evidence (GRADE)	Comments
**Live-birth** Live-birth Follow-up: 6 months	**Study population**		**RR 0.97** (0.81 to 1.17)	332 (2 studies)	⊕⊕⊕⊖ **moderate**[1]	
	**586 per 1000**	**568 per 1000** (474 to 685)				
	**Moderate**					
	**586 per 1000**	**568 per 1000** (475 to 686)				
**Miscarriage** Miscarriage Follow-up: 6 months	**Study population**		**RR 0.94** (0.41 to 2.16)	332 (2 studies)	⊕⊕⊕⊖ **moderate**[1]	
	**65 per 1000**	**61 per 1000** (27 to 141)				
	**Moderate**					
	**65 per 1000**	**61 per 1000** (27 to 140)				
**Pregnancy** Serum β-HCG laboratory examination; ultrasonography of uterus Follow-up: 6 months	**Study population**		**RR 0.97** (0.83 to 1.14)	332 (2 studies)	⊕⊕⊕⊖ **moderate**[1]	
	**651 per 1000**	**631 per 1000** (540 to 742)				
	**Moderate**					
	**651 per 1000**	**631 per 1000** (540 to 742)				
**Ovulation-induction** The serum progesterone laboratory examination Follow-up: 6 months	**Study population**		**RR 0.99** (0.86 to 1.14)	332 (2 studies)	⊕⊕⊕⊖ **moderate**[1]	
	**710 per 1000**	**703 per 1000** (611 to 809)				
	**Moderate**					
	**710 per 1000**	**703 per 1000** (611 to 809)				

*The basis for the **assumed risk** (e.g. the median control group risk across studies) is provided in footnotes. The **corresponding risk** (and its 95% confidence interval) is based on the assumed risk in the comparison group and the **relative effect** of the intervention (and its 95% CI).

CI: Confidence interval; **RR:** Risk ratio;

GRADE Working Group grades of evidence
High quality: Further research is very unlikely to change our confidence in the estimate of effect.
Moderate quality: Further research is likely to have an important impact on our confidence in the estimate of effect and may change the estimate.
Low quality: Further research is very likely to have an important impact on our confidence in the estimate of effect and is likely to change the estimate.
Very low quality: We are very uncertain about the estimate.

[1] Downgraded one level for serious risk of performance bias.

Met-CC compared to LOD-CC for CCR-PCOS

Patient or population: patients with CCR-PCOS
Settings: Inpatients
Intervention: Met-CC
Comparison: LOD-CC

Outcomes	Illustrative comparative risks* (95% CI) Assumed risk LOD-CC	Corresponding risk Met-CC	Relative effect (95% CI)	No of Participants (studies)	Quality of the evidence (GRADE)	Comments
**Live-birth** Live-birth Follow-up: 6 months	**Study population**		**RR 1.43** (0.57 to 3.57)	28 (1 study)	⊕⊖⊖⊖ **very low**[1,2]	
	**350 per 1000**	**500 per 1000** (199 to 1000)				
	**Moderate**					
	**350 per 1000**	**500 per 1000** (199 to 1000)				
**Miscarriage** Miscarriage Follow-up: 6 months	**Study population**		**RR 1.00** (0.24 to 4.14)	28 (1 study)	⊕⊖⊖⊖ **very low**[1,2]	
	**250 per 1000**	**250 per 1000** (60 to 1000)				
	**Moderate**					
	**250 per 1000**	**250 per 1000** (60 to 1000)				
**Pregnancy** Serum β-HCG laboratory examination; ultrasonography of uterus Follow-up: 6 months	**Study population**		**RR 1.25** (0.73 to 2.14)	28 (1 study)	⊕⊖⊖⊖ **very low**[1,2]	
	**600 per 1000**	**750 per 1000** (438 to 1000)				
	**Moderate**					
	**600 per 1000**	**750 per 1000** (438 to 1000)				
**Ovulation-induction** The serum progesterone laboratory examination Follow-up: 6 months	**Study population**		**RR 1.25** (0.85 to 1.84)	28 (1 study)	⊕⊖⊖⊖ **very low**[1,2]	
	**700 per 1000**	**875 per 1000** (595 to 1000)				
	**Moderate**					
	**700 per 1000**	**875 per 1000** (595 to 1000)				

*The basis for the **assumed risk** (e.g. the median control group risk across studies) is provided in footnotes. The **corresponding risk** (and its 95% confidence interval) is based on the assumed risk in the comparison group and the **relative effect** of the intervention (and its 95% CI).

CI: Confidence interval; **RR:** Risk ratio;

GRADE Working Group grades of evidence
High quality: Further research is very unlikely to change our confidence in the estimate of effect.
Moderate quality: Further research is likely to have an important impact on our confidence in the estimate of effect and may change the estimate.
Low quality: Further research is very likely to have an important impact on our confidence in the estimate of effect and is likely to change the estimate.
Very low quality: We are very uncertain about the estimate.

[1] Downgraded two levels for very serious risk of selection bias: unclear about random sequence generation and allocation concealment.
[2] Downgraded two levels for very serious imprecision: Broad confidence interval, very few events.
