# Supplementary material for: Metformin With or Without Clomiphene Citrate Versus Laparoscopic Ovarian Drilling With or Without Clomiphene Citrate to Treat Patients With Clomiphene Citrate-Resistant Polycystic Ovary Syndrome: A Systematic Review and Meta-Analysis
Source: Front Pharmacol. 2022 Jun 22;13:576458. doi: 10.3389/fphar.2022.576458 (PMC9256960; doi:10.3389/fphar.2022.576458)
Supplement: Supplementary file 2 [file DataSheet1.PDF]

Metformin with or without clomiphene citrate versus laparoscopic ovarian drilling with or without clomiphene citrate to treat patients with clomiphene citrate-resistant polycystic ovary syndrome: A systematic review and meta-analysis

Ming-Li Sun[1], Wen-Pei Bai[2], Qing-Kun Song[3], Hui-Ying Wang[2], Guo-Lan Gao[4*], Liang Zheng[5*], Xing-He Wang[1*]

Supplementary

Content

Appendix 1: Protocol ……..……..…….……..…...……………..…………..…………1

Appendix 2: Search strategy ……………………..……………………………………4

Appendix 3: Sensitivity analysis …………………...………………………………6

Appendix 4: Meta-regression analysis ...…………. …………………… ….………7

Appendix 5: Subgroup analysis ...……………………………………… ….………8

Appendix 6: Meta-analysis reperformed after exclusion of Hashim HA. (2011) ………9

Appendix 7: Summary of findings …………………………………..…..………10

Appendix 1: Protocol

1. **Background**: Which is optimal to treat clomiphene citrate-resistant polycystic ovary syndrome (CCR-PCOS) with LOD or metformin remains a problem. There are three inconsistent or even contradictory views.

2. **Objective**: The present meta-analysis aimed to evaluate the effectiveness and safety of Metformin with or without CC and to compare them with LOD with or without CC (Met/Met-CC vs. LOD/LOD-CC) in women with CCR-PCOS who also have anovulation.

3. **Methods:**

1) **Information sources and search strategy:** PubMed, Embase, and the Cochrane Central Register of RCTs are searched for articles published. The reference lists of the included articles are scanned for additional relevant studies. Grey (unpublished) literature is identified by searching the websites of clinical practice guideline collections, clinical trial registries, national and international medical specialty societies, and recent conference abstracts. Full-text articles of potentially relevant studies that are unavailable through the university library are requested from the authors. In addition, searched the Open Grey database (https://opengrey.eu/), Grey Literature Report (GreyLit Network) (http://www.greylit.org/), the preprint database MedRxiv (https://www.medrxiv.org/) are searched to identify published, ongoing, and unpublished research reports, doctoral dissertations, some conference papers, some official publications, and other types of grey literature.

2) **Eligibility criteria and study selection**

Two reviewers independently screened the titles and abstracts to determine whether the articles were relevant to the meta-analysis based on the pre-defined inclusion criteria. The full texts of potentially eligible studies are then reviewed before the final selection. Any disagreement is resolved in consultation with the third author.

The inclusion are as follows: (1) study population of patients with both CCR-PCOS and anovulation-related infertility; (2) intervention of LOD-controlled Met treatment despite continuing CC; (3) reporting of fertility outcomes (ovulation,

pregnancy, and live-birth rates); (4) RCT study design.

The exclusion criteria are as follows: (1) duplicates; (2) study design other than RCT (e.g., reviews, meta-analyses, case reports, guidelines, trial protocols); (3) absence of comparison between Met/Met-CC and LOD/LOD-CC; (4) absence of fertility outcomes.

3) **Data extraction**: If available, the following information was extracted from each article: study period, inclusion, and exclusion criteria, first author, year of publication, subjects' country of residence, definition of CCR, number of participants in each group, clinical characteristics of the participants, treatment regimens, duration of treatment, follow-up period, and fertility outcomes (ovulation, pregnancy, and live-birth rates).
4) **Assessment of risk of bias**: Two researchers independently conducted quality assessment of all the included articles using the Cochrane risk-of-bias tool in the following domains: selection bias, performance bias, detection bias, attrition bias, reporting bias, and other bias. Each domain was classified as low, unclear, or high risk. If there are discrepancies, the final assessment decision is made in consultation with the third researcher.
5) **Data synthesis**: pooling of the data form forest plots.
6) **Investigation of heterogeneity**: Heterogeneity among the included studies is analyzed using the I^2 test, as follows: $I^2 = [(Q–df)/Q] \times 100\%$, where Q is the χ^2 heterogeneity statistic and df is the degrees of freedom. I^2 values > 75% indicate high heterogeneity, whereas values between 50% and 75% indicate moderate heterogeneity. I^2 values between 25% and 50% indicate low heterogeneity, and values below 25% indicate no heterogeneity.
7) **Sensitivity analysis**: Sensitivity analysis is performed using the "random-effects model" and "leave-one-out" methods.
8) **Meta-regression analysis**: If any heterogeneity occurred, the reasons for it are ascertained using a meta-regression analysis of the primary effectiveness outcome.
9) **Subgroup analysis**: Subgroup analysis of the primary effectiveness outcome is

also performed to explain expected significant heterogeneity.

10) **Assessment of reporting biases**: Potential publication bias was examined using the funnel plot, Egger linear regression, and Begg rank correlation tests.

11) Overall quality of the body of evidence: "Summary of findings" table

The overall quality of this meta-analysis is estimated according to the GRADE four-step approach.

12) **Statistics and statistical software**: The statistical significance level was set at $p < 0.05$. In the meta-analysis, Review Manager 5.3 and one of the Cochrane Collaboration Tools were used to create the risk-of-bias graph; the statistical software package Stata16 (Stata Corp., College Station, TX, USA), and GRADEpro 3.6 software were also used.

4. **The expected results**: We expect to quantify the effectiveness and safety size of Metformin with or without CC and LOD with or without CC (Met/Met-CC vs. LOD/LOD-CC) in women with CCR-PCOS who also have anovulation.

Appendix 2: Search strategy

Cochrane

ID	Search	Hits
#1	(polycystic ovarian syndrome) OR (polycystic ovarian disease) OR PCOS OR PCOD with Cochrane Library publication date Between Jan 1966 and Aug 2019	3167
#2	metformin OR dimethylguanylguanidine OR dimethylbiguanidine OR glucophage OR dimethylbiguanide OR dmbg with Cochrane Library publication date Between Jan 1966 and Aug 2019	9247
#3	#1 and #2	980
#4	(laparoscopic ovarian drilling) OR (laparoscopic ovarian diathermy) OR LOD OR (laparoscopic ovarian electrocautery) OR LOE with Cochrane Library publication date Between Jan 1966 and Aug 2019	761
#5	#3 and #4	31
#6	ovulation OR pregnancy OR live-birth with Cochrane Library publication date Between Jan 1966 and Aug 2019	52455
#7	#5 and #6	28
#8	(randomized OR randomly OR trial OR placebo) with Cochrane Library publication date Between Jan 1966 and Aug 2019	880092
#9	#7 and #8	26
#10	animal NOT human with Cochrane Library publication date Between Jan 1966 and Aug 2019	6029
#11	#9 NOT #10	26
17 Trials matching "#11 - #9 NOT #10"<br>8 Cochrane Reviews matching "#11 - #9 NOT #10"<br>1 Clinical Answer matching "#11 - #9 NOT #10"<br>0 Cochrane Protocols matching "#11 - #9 NOT #10"<br>0 Editorials matching "#11 - #9 NOT #10"<br>0 Special Collections matching "#11 - #9 NOT #10"		

Embase Session Results

No.	Query Results	Results
#1	(polycystic AND ovarian AND ('syndrome'/exp OR syndrome) OR (polycystic AND ('ovary'/exp OR ovary) AND ('syndrome'/exp OR syndrome)) OR (polycystic AND ovarian AND ('disease'/exp OR disease)) OR pcos OR pcod) AND [1966-2019]/py	27,431
#2	(metformin OR dimethylguanylguanidine OR dimethylbiguanidine OR glucophage OR dimethylbiguanide OR dmbg) AND [1966-2019]/py	64,141
#3	#1 AND #2	3,636
#4	(laparoscopic AND ovarian AND drilling OR (laparoscopic AND ovarian AND diathermy) OR lod OR (laparoscopic AND ovarian AND electrocautery) OR loe) AND [1966-2019]/py	27,990
#5	#3 AND #4	162
#6	(ovulation OR pregnancy OR 'live birth') AND [1966-2019]/py	956,080

#7	#5 AND #6	148
#8	(randomized AND controlled AND trial OR (random AND allocation)) AND [1966-2019]/py	836,654
#9	#7 AND #8	39
#10	animal NOT human AND [1966-2019]/py	4,417,880
#11	#9 NOT #10	39
39 results for search #11		

PubMed

Query	Results
((polycystic ovarian syndrome) OR (polycystic ovary syndrome) OR (polycystic ovarian disease) OR PCOS OR PCOD) AND (metformin OR dimethylguanylguanidine OR dimethylbiguanidine OR glucophage OR dimethylbiguanide OR dmbg) AND ((laparoscopic ovarian drilling) OR (laparoscopic ovarian diathermy) OR LOD OR (laparoscopic ovarian electrocautery) OR LOE) AND (ovulation OR pregnancy OR live-birth) AND ((randomized controlled trial) OR (random allocation)) Filters: from 1966/1/1 - 2019/8/31	15
15 results	

Appendix 3: Sensitivity analysis

figure 1. Sensitivity analysis of live birth/ongoing pregnancy rate in the Met/Met-CC vs. LOD/LOD-CC comparison using (A) the random-effect model and (B) the leave-one-out method.

Notes: Met = metformin, LOD = laparoscopic ovarian drilling. In figure (B), 1 = Hashim HA. (2011); 2 = Hamed HO. (2010); 3 = Palomba S. (2010); 4 = Palomba S. (2005); 5 = Palomba S. (2004).

Appendix 4: Meta-regression analysis for live-birth/ongoing-pregnancy rate

```
. metareg _ES, wsse(_selogES) bsest(reml)

Meta-regression                                       Number of obs  =       5
REML estimate of between-study variance               tau2           =   .0863
% residual variation due to heterogeneity             I-squared_res  =  58.58%
With Knapp-Hartung modification
```

_ES	Coef.	Std. Err.	t	P>|t|	[95% Conf.	Interval]
_cons	1.097747	.1782598	6.16	0.004	.6028182	1.592675

```
. metareg _ES treatment, wsse(_selogES) bsest(reml)

Meta-regression                                       Number of obs  =       5
REML estimate of between-study variance               tau2           =   .1356
% residual variation due to heterogeneity             I-squared_res  =  64.87%
Proportion of between-study variance explained        Adj R-squared  = -57.13%
With Knapp-Hartung modification
```

_ES	Coef.	Std. Err.	t	P>|t|	[95% Conf.	Interval]
treatment	.0291875	.3173433	0.09	0.933	-.9807404	1.039115
_cons	1.016417	.8915663	1.14	0.337	-1.820945	3.853779

```
. metareg _ES followup, wsse(_selogES) bsest(reml)

Meta-regression                                       Number of obs  =       5
REML estimate of between-study variance               tau2           =  .07298
% residual variation due to heterogeneity             I-squared_res  =  59.84%
Proportion of between-study variance explained        Adj R-squared  =  15.43%
With Knapp-Hartung modification
```

_ES	Coef.	Std. Err.	t	P>|t|	[95% Conf.	Interval]
followup	.6594765	.4894209	1.35	0.271	-.8980791	2.217032
_cons	-.1300647	.926921	-0.14	0.897	-3.079941	2.819811

```
. metareg _ES country, wsse(_selogES) bsest(reml)

Meta-regression                                       Number of obs  =       5
REML estimate of between-study variance               tau2           =  .06372
% residual variation due to heterogeneity             I-squared_res  =  40.43%
Proportion of between-study variance explained        Adj R-squared  =  26.17%
With Knapp-Hartung modification
```

_ES	Coef.	Std. Err.	t	P>|t|	[95% Conf.	Interval]
country	.4672905	.323961	1.44	0.245	-.5636981	1.498279
_cons	.3924541	.5153649	0.76	0.502	-1.247667	2.032575

```
. metareg _ES sample, wsse(_selogES) bsest(reml)

Meta-regression                                       Number of obs  =       5
REML estimate of between-study variance               tau2           =    .151
% residual variation due to heterogeneity             I-squared_res  =  64.51%
Proportion of between-study variance explained        Adj R-squared  = -75.00%
With Knapp-Hartung modification
```

_ES	Coef.	Std. Err.	t	P>|t|	[95% Conf.	Interval]
sample	-.0006242	.0022384	-0.28	0.798	-.0077477	.0064993
_cons	1.182602	.378261	3.13	0.052	-.0211931	2.386398

Appendix 5: Subgroup analysis

figure 2. Subgroup analysis of Met/Met-CC vs. LOD/LOD-CC based on (A) Treatment method (B) follow-up period after pregnancy, and (C) countries of participants.

Appendix 6: Meta-analysis reperformed after exclusion of Hashim HA. (2011)

figure 3. Forest plot of effectiveness and safety outcomes comparison the Met/Met-CC with LOD/LOD-CC using the fixed-effect model: (A) effectiveness; (B) safety.

Notes: Met = metformin, CC = clomiphene citrate, LOD = laparoscopic ovarian drilling.

figure 4. Sensitivity analysis of live birth/ongoing pregnancy rate in the Met/Met-CC vs. LOD/LOD-CC comparison using (A) the random-effect model; (B) the leave-one-out method.

Notes: Met = metformin, LOD = laparoscopic ovarian drilling. L = live birth/ongoing pregnancy; L = live birth/ongoing pregnancy rate; P = pregnancy; O = ovulation induction.

figure 5. Funnel plot of comparative (A) live-birth/ongoing-pregnancy rate, (B) pregnancy rate, (C) ovulation-induction rate, and (D) miscarriage rate.

Appendix 7: Summary of findings for the main comparison. Met/Met-CC compared to LOD/LOD-CC.
